# Evolution of ribonuclease H genes in prokaryotes to avoid inheritance of redundant genes

**DOI:** 10.1186/1471-2148-7-128

**Published:** 2007-07-31

**Authors:** Hiromi Kochiwa, Masaru Tomita, Akio Kanai

**Affiliations:** 1 Institute for Advanced Biosciences, Keio University, Tsuruoka 997-0017, Japan; 2 Systems Biology Program, Graduate School of Media and Governance, Keio University, Fujisawa 252-8520, Japan

## Abstract

**Background:**

A theoretical model of genetic redundancy has proposed that the fates of redundant genes depend on the degree of functional redundancy, and that functionally redundant genes will not be inherited together. However, no example of actual gene evolution has been reported that can be used to test this model. Here, we analyzed the molecular evolution of the ribonuclease H (RNase H) family in prokaryotes and used the results to examine the implications of functional redundancy for gene evolution.

**Results:**

In prokaryotes, RNase H has been classified into RNase HI, HII, and HIII on the basis of amino acid sequences. Using 353 prokaryotic genomes, we identified the genes encoding the RNase H group and examined combinations of these genes in individual genomes. We found that the RNase H group may have evolved in such a way that the RNase HI and HIII genes will not coexist within a single genome – in other words, these genes are inherited in a mutually exclusive manner. Avoiding the simultaneous inheritance of the RNase HI and HIII genes is remarkable when RNase HI contains an additional non-RNase H domain, double-stranded RNA, and an RNA-DNA hybrid-binding domain, which is often observed in eukaryotic RNase H1. This evolutionary process may have resulted from functional redundancy of these genes, because the substrate preferences of RNase HI and RNase HIII are similar.

**Conclusion:**

We provide two possible evolutionary models for RNase H genes in which functional redundancy contributes to the exclusion of redundant genes from the genome of a species. This is the first empirical study to show the effect of functional redundancy on changes in gene constitution during the course of evolution.

## Background

The science of molecular evolution has paved the way for understanding evolutionary processes in which genetic redundancy (the presence of two or more genes capable of serving the same functional role in an organism) is a major source of genetic novelty and robustness. In fact, a recent analysis of 106 bacterial genomes revealed that a significant number of genetic redundancies have persisted in individual genomes [1], and systematic gene deletion experiments have demonstrated that approximately 300 out of 4000 genes are indispensable for two bacterial species, *Escherichia coli *[2] and *Bacillus subtilis *[3], suggesting the presence of considerable redundancy in the bacterial genome. However, these findings also raise the question of how genetic redundancy is maintained within a genome, because functionally redundant genes are likely to be eliminated by selective pressure as shown in a large-scale analysis of protein-protein interactions in *Saccharomyces cerevisiae*, in which the redundant interactions due to duplicated genes generally are not persisted long after gene duplication [4]. In order to explain the process, a theoretical model has been developed to provide insight into the retention of redundant genes, which is hypothesized to depend on their degree of functional redundancy [5], there have been no empirical studies of gene evolution to support this theoretical model directly. The study described here aimed to substantiate the model of evolution of genetic redundancy on the basis of the analysis of a ribonuclease family that has contributed to our understanding of some aspects of molecular evolution, such as adaptive evolution [6,7], positive Darwinian selection [8], and the origin of retroviruses with long terminal repeats (LTR) [9].

Ribonuclease H (RNase H; EC 3.1.26.4), one member of the ribonuclease family, is an enzyme that specifically degrades the RNA moiety of RNA-DNA hybrids [10]. Because various studies have revealed the presence of RNase H in eukaryotes, prokaryotes, and retroviruses, this compound is considered to be one of the most widely conserved enzymes [11]. Although the physiological functions of RNase H are not fully understood, this enzyme is thought to play several roles in DNA replication [12-14], DNA repair [15,16], and RNA transcription [17,18]. In terms of its medical importance, RNase H activity in retroviruses (including HIV-1) is necessary for their replication, and the enzyme has thus been regarded as one of the drug targets for AIDS chemotherapy [19]. This enzyme is also suggested to be related to the antiviral immune response in humans, because mutations of the RNase H-encoding gene have been found in individuals affected by a human neurological disease, Aicardi-Goutières syndrome [20]. Therefore, the accumulation of experimental data on the secondary structures and enzymatic features of RNase H from many studies of its biological significance has provided us with an opportunity to use this knowledge in the field of molecular evolution.

Unlike retroviruses, which possess a single RNase H gene, most prokaryotic and eukaryotic genomes contain multiple RNase H genes. According to the nomenclature for these enzymes, prokaryotic RNase H is generally classified into three groups: RNase HI, HII, and HIII. Eukaryotic RNase H is divided into RNase H1 and H2 [21]. Phylogenetic analyses using RNase H sequences have proposed the following classification: Type 1 (prokaryotic RNase HI, eukaryotic RNase H1, and viral RNase H) and Type 2 (prokaryotic RNase HII and HIII, and eukaryotic RNase H2), and it is important to note that no prokaryotic species with a combination of RNase HI and HIII genes has yet been identified [11]. Additionally, in contrast to eukaryotes, which tend to contain both RNase H1 and H2 genes, the combination of RNase H genes in prokaryotes varies among species, and the overall nature of this variation is poorly understood. Therefore, further study is required to clarify the presence or absence of RNase H genes in these species.

We conducted a comparative analysis of the complete genomes of 353 prokaryotes (326 bacteria and 27 archaea) and examined the combination of RNase H genes and the potential evolutionary processes that could explain the effects of functional redundancy on gene evolution, as described by a theoretical model of genetic redundancy [5]. Our findings suggest that the RNase HI and HIII genes have evolved in a mutually exclusive manner owing to their functional similarities. This molecular evolution of RNase H genes is the first actual example of how the degree of functional redundancy has implications for changes in gene constitution during the course of evolution.

## Results

### Genome-wide identification of RNase H genes

To identify RNase HI, HII, and HIII coding sequences in a genome, two strategies (a remote homology search and a protein domain search) were applied to ensure maximum coverage of the genes (See Methods). Using the complete genomes for 326 strains from 235 bacterial species and 27 strains from 27 archaeal species, we retrieved 342 RNase HI genes, 333 RNase HII genes, and 76 RNase HIII genes (see Additional file 1). Almost all genomes contained one or more RNase H genes, and there was little difference in the types and numbers of RNase H genes among several strains of a given species, with the exception of *Buchnera aphidicola *and *Xanthomonas campestris*. The RNase HI-related gene of *B. aphidicola *str. APS (*Acyrthosiphon pisum*) contained a frameshift mutation that resulted in a loss of RNase H activity (Dr. Naoto Ohtani, Keio University, personal communication), whereas non-frameshifted RNase HI genes were identified in *B. aphidicola *str. Bp (*Baizongia pistaciae*) and *B. aphidicola *str. Sg (*Schizaphis graminum*). A frameshift mutation was also found in the RNase HII-related gene of *Xanthomonas campestris *pv. *vesicatoria *str. 85-10. In contrast, other strains of *X. campestris *pv. *campestris *(str. 8004 and str. ATCC 33913) had a non-frameshifted RNase HII. Therefore, we assumed that *B. aphidicola *had an RNase HI gene and *X. campestris *had an RNase HII gene at the species level. Accordingly, we counted the number of RNase H genes in the 27 archaeal and 235 bacterial species listed in Table 1. The RNase HI gene was present in 33% (9/27) of the archaeal species and 89% (210/235) of the bacterial species, the RNase HII gene was present in all archaeal species and in 94% (220/235) of the bacterial species, and the RNase HIII gene was identified in 4% (1/27) of the archaeal species and in 17% (40/235) of the bacterial species. This result is consistent with a previous report that RNase HII is the more universal gene in prokaryotes [11]. Most species had a single copy of a given gene, but multiple genes encoding RNase HI were found in 11% (3/27) of the archaeal species and 16% (37/235) of the bacterial species.

**Table 1:** Number of RNase H genes identified in the archaeal and bacterial species.

Kingdom	Type	No. of species	No. of RNase H genes		
			
			1	2	3
Archaea	RNase HI	9	6	3	0
	RNase HII	27	27	0	0
	RNase HIII	1	1	0	0
					
Bacteria	RNase HI	210	173	32	5
	RNase HII	220	219	1	0
	RNase HIII	40	40	0	0

### Alteration of RNase H combinations in closely related species

**Figure 1:**
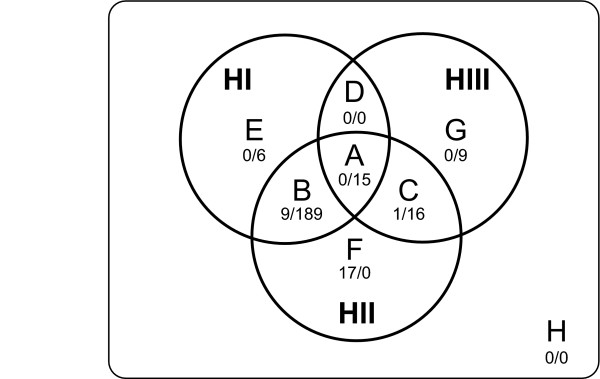
**Potential combinations of the three RNase H genes**. The Venn diagram consists of three circles (HI, RNase HI; HII, RNase HII; HIII, RNase HIII) that represent the possible combinations of RNase H genes as Groups A to H. The 353 prokaryotic genomes were classified according to this system, and the results are presented in Additional File 1. The numbers under Groups A to H indicate the number of archaeal (left) and bacterial (right) species identified in each of the groups.

Contrary to the situation in eukaryotes, in which both RNase H1 and RNase H2 tend to coexist, various combinations of RNase H genes have been found in prokaryotes [11]. To compare the presence and absence of RNase H genes among prokaryotes, we examined the combinations of RNase H genes in individual genomes. Three types of RNase H genes can theoretically produce eight combinations of genes, as shown in the Venn diagram in Figure 1. Because, in practice, we found no species that lacked all three genes (Group H), all species were classified on the basis of the remaining seven RNase H combinations (Table 2). No prokaryotic genome contained the combination of only RNase HI and HIII (Group D); this supports the results of a previous study [11] at the genome-wide level. Although many archaeal species contained only the RNase HII gene (Group F) or a combination of RNase HI and HII genes (Group B) – a finding in agreement with previous reports [22,23] – one of the euryarchaeota, *Methanosphaera stadtmanae *DSM 3091, combined RNase HII with RNase HIII (Group C) instead. On the other hand, 189 of the 235 bacterial species (80%) had combinations of the RNase HI and HII genes (Group B) and 16 of the 235 species (7%) had combinations of the RNase HII and HIII genes (Group C). At the same time, the RNase H combinations in bacteria exhibited more variety than those in the archaea and seemed to differ even among related species, especially in the firmicutes. Interestingly, species that had all three RNase H genes (Group A) were limited to the firmicutes.

**Table 2:** Combinations of the three RNase H genes found in the archaeal and bacterial genomes.

Kingdom	Subdivision	No. of species	Classification of RNase H combination						
			
			Group A	Group B	Group C	Group D	Group E	Group F	Group G
Archaea	Crenarchaeota	5	0	3	0	0	0	2	0
	Euryarchaeota	21	0	6	1	0	0	14	0
	Nanoarchaeota	1	0	0	0	0	0	1	0
	Total	27	0	9	1	0	0	17	0
									
Bacteria	Acidobacteria	1	0	1	0	0	0	0	0
	Actinobacteria	20	0	18	1	0	1	0	0
	Aquificae	1	0	0	1	0	0	0	0
	Bacteroidetes	4	0	4	0	0	0	0	0
	Chlamydiae	7	0	0	7	0	0	0	0
	Chlorobi	3	0	3	0	0	0	0	0
	Chloroflexi	2	0	2	0	0	0	0	0
	Cyanobacteria	8	0	8	0	0	0	0	0
	Deinococcus-Thermus	3	0	3	0	0	0	0	0
	Firmicutes	49	15	18	7	0	0	0	9
	Fusobacteria	1	0	1	0	0	0	0	0
	Planctomycetes	1	0	0	0	0	1	0	0
	Alphaproteobacteria	38	0	38	0	0	0	0	0
	Betaproteobacteria	23	0	23	0	0	0	0	0
	Deltaproteobacteria	11	0	11	0	0	0	0	0
	Epsilonproteobacteria	5	0	5	0	0	0	0	0
	Gammaproteobacteria	52	0	49	0	0	3	0	0
	Spirochaetes	5	0	4	0	0	1	0	0
	Thermotogae	1	0	1	0	0	0	0	0
									
	Total	235	15	189	16	0	6	0	9

All species were classified on the basis of the RNase H combinations shown in the Venn diagram in Figure 1.

**Figure 2:**
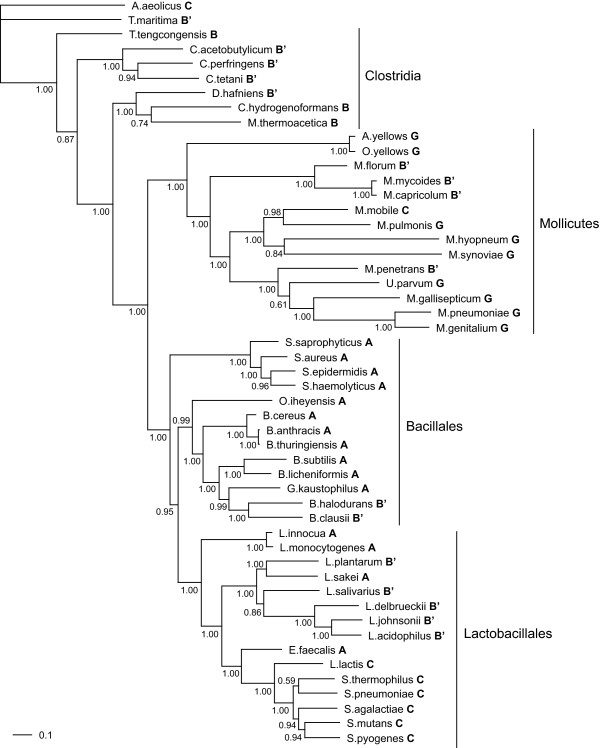
**Bayesian phylogenetic tree of 49 species in the firmicutes**. A phylogenetic tree was constructed with Bayesian inference on the basis of the *gyrB *sequence alignments of 49 species in the firmicutes. Numbers at the nodes represent posterior probabilities. The scale bar represents 0.1 substitutions per site. Letters A through G next to the species names represent the combinations of RNase H genes defined in Figure 1; B' indicates the presence of dsRHbd.

To elucidate the relationship between RNase HI and HIII, the evolutionary genomic constitution of the RNase H genes was examined in 49 species of firmicutes, because RNase HIII is especially common in this group (classes A, C, D, and G in Table 2). First, we constructed a Bayesian tree based on the nucleotide sequences of the DNA gyrase subunit B (*gyrB*) genes of the firmicutes, which have been used to infer phylogenetic relationships among prokaryotes [24], and displayed the RNase H combinations of each species (Figure 2). The results of our phylogenetic analysis indicate that RNase H combinations differed even among closely related species. For example, the species in the mollicutes were classified into Groups B (RNase HI and HII), C (RNase HII and HIII), and G (only RNase HIII), showing that the RNase HIII gene is not found in the mollicutes that retain the RNase HI gene. In addition, species with all three RNase H genes (Group A) were found only in the bacillales and lactobacillales, because this combination is not found in species other than firmicutes (see Table 2).

More noteworthy is the fact that the RNase HI genes of the species that also have RNase HII genes (Group B) often encode additional conserved protein domains, as represented by the presence of Group B' in Figure 2. This non-RNase H domain was first identified in the N-terminal portion of eukaryotic RNase H1 [25] and was designated as a double-stranded RNA (dsRNA) and an RNA-DNA hybrid-binding domain (dsRHbd) because of its ability to bind to dsRNA as well as RNA-DNA hybrids [26-28]. In prokaryotes, it has been reported that RNases HI of *Bacillus halodurans *[29] and of *Shewanella *sp. SIB1 [30] that have dsRHbd in the N-terminus possess RNase H activity. In contrast, no such domain was identified in RNase HI of the species that had all three types of RNase H (Group A). Interestingly, RNase HI of *B. subtilis *[REFSEQ: NP_390082], a member of Group A, exhibited neither RNase H activity nor other nuclease activity, even though RNase HII and HIII possess RNase H activity [22,31]. This may indicate a difference of RNase H activity between RNase HI without dsRHbd (Group A) and RNase HI with dsRHbd (Group B').

**Figure 3:**
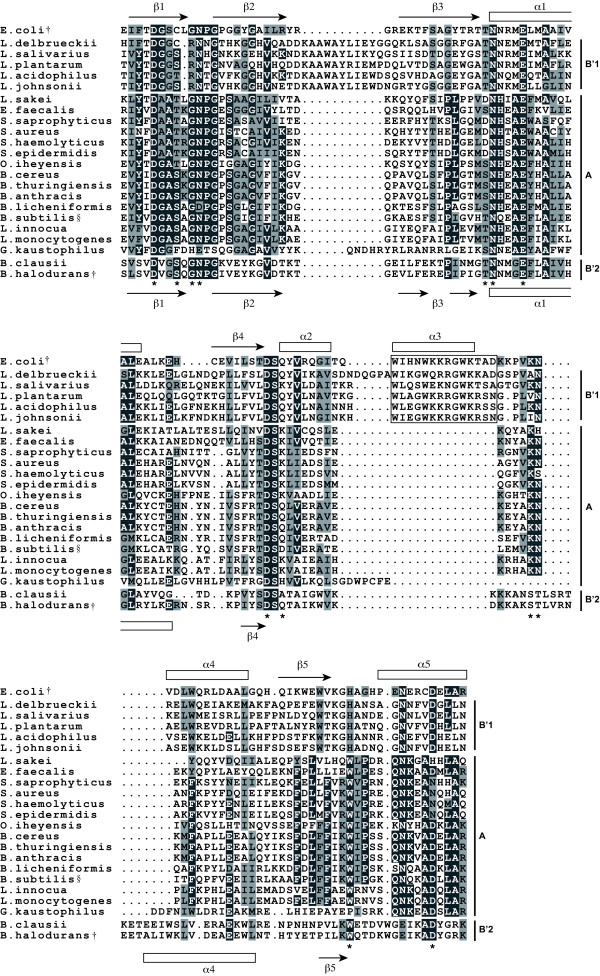
**Amino acid sequence alignments for the RNase HI domains**. RNase HI protein sequences were derived from the bacillales and lactobacillales listed in Additional File 2 and were aligned by using the Clustal method. Arrows and rectangles indicate beta-strands and alpha-helices, respectively. The upper and lower secondary structures were generated on the basis of the RNase HI domains of *E. coli *[33] and *B. halodurans *[29]. Dark and light shadings indicate highly conserved and similar amino acid residues, respectively. Asterisks denote amino acid residues that are involved in the catalytic function of RNase HI. The boxed region below the label for alpha-helix 3 forms a basic protrusion handle in the *E. coli *RNase HI structure. The combinations of RNase H genes are represented to the right of the sequences (see the text for details). The symbols ^† ^and ^§ ^indicate active and inactive RNase H, respectively.

To identify differences in the primary and secondary structures between RNase HI without dsRHbd (Group A) and RNase HI with dsRHbd (Group B'), multiple alignments were performed using the amino acid sequences of each RNase HI domain in the bacillales and lactobacillales and the *E. coli *RNase HI domain. If the species had multiple RNase HI genes, one gene that was more similar to *E. coli *RNase HI than to any other gene was selected; these are described in Additional file 2. As a result, the RNase HI sequences were divided into three groups (Figure 3). The amino acid sequences of RNase HI in Group A were similar to that of *B. subtilis *RNase HI, which exhibited no nuclease activity. In contrast, the primary structures of RNase HI with dsRHbd formed two groups: Group B'1, in which the primary structures of lactobacillales RNase HI were similar to that of *E. coli *RNase HI, whose nuclease activity has been demonstrated [32], and Group B'2, in which *B. halodurans *and *B. clausii *RNase HI had little similarity to other RNase HI but showed RNase H activity [29]. There is also a marked difference in the secondary structure. RNase HI in Group A lacked the basic protrusion handle (alpha-helix 3) involved in substrate binding of *E. coli *RNase HI [33,34]. On the other hand, all of the lactobacillales RNase HI with dsRHbd in Group B'1 had the basic protrusion handle. Although the basic protrusion handle is not observed in *B. halodurans *and *B. clausii *(Group B'2), it has been proposed that dsRHbd could functionally compensate for this basic protrusion [29]. From the relationship between structural similarity and RNase H activity, it can be inferred that RNase HI with dsRHbd in Group B' exhibits RNase H activity but it is unclear whether RNase HI in Group A exhibits RNase H activity or not, because the archaeal RNase HI of *Halobacterium *sp. NRC-1 [22] and *Sulfolobus tokodaii *7 [23] exhibited weak RNase H activity despite the absence of the basic protrusion handle. However, the fact that a double knockout of RNase HII and HIII genes in *B. subtilis *yields a lethal phenotype [31] indicates that Group A RNase HI genes encoded in the *B. subtilis *genome do not have the ability to compensate for functions of RNase HII and HIII. Therefore, our results (that RNase HIII is not present in Group B but is present in Group A) suggest that there is some sort of relationship between protein functions and gene constitutions.

### Phylogenetic distribution of dsRHbd sequences

Our results (Table 2) clearly showed that the combination of RNase HI and HIII genes (Group D) was not found in the prokaryotes and that most bacterial species had combinations of RNase HI and HII (Group B) or RNase HII and HIII (Group C). Moreover, the combination of RNase H genes has been altered even among closely related species in such a way that functional RNase HI and HIII genes do not coexist in a single genome; in other words, our results provide evidence that RNase HI and HIII tend to evolve in a mutually exclusive manner. Avoiding the simultaneous inheritance of the RNase HI and HIII genes is remarkable when RNase HI contains dsRHbd in the firmicutes, because dsRHbd sequences were found in 15 out of 18 species that combined the RNase HI and HII genes (Group B) and were not found in any of the 15 species that had all three RNase H genes (group A) (see Figure 2). Therefore, dsRHbd appears to be a key domain in the evolutionary process that has led to the current distribution of RNase H genes. Although the characteristics of dsRHbd, such as its enzymatic features [25,27] and its secondary structure, have been compared with those of eukaryotic RNase HI [35], little is known about the number and types of dsRHbd in prokaryotes. Therefore, we searched for dsRHbd sequences in the complete genomes of 326 strains from 235 bacterial species and 27 strains from 27 archaeal species in the same way that we searched for the RNase H sequences (See Methods).

**Figure 4:**
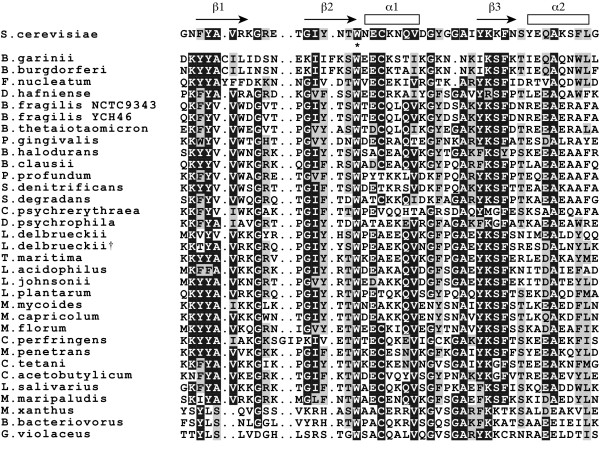
**Amino acid sequence alignments of dsRHbds from prokaryotes**. Arrows and rectangles indicate beta-strands and alpha-helices, respectively. These secondary structures were generated on the basis of the N-terminus domain of *S. cerevisiae *RNase H1 [35]. Arrows and rectangles indicate beta-strands and alpha-helices, respectively. Dark and light shadings indicate highly conserved and similar amino acid residues, respectively. Asterisk represents an identical amino acid residue. The symbol ^† ^indicates that the gene containing the resolvase domain encodes the amino acid sequence of dsRHbd. For detailed information, see Table 3.

The results revealed that the genomes of 30 bacterial species (one of which had two strains) and 1 archaeal species encoded dsRHbd (Table 3), and that the distribution pattern of dsRHbd in prokaryotes did not appear to be correlated with the phylogenetic pattern. Most dsRHbds are fused with the RNase HI domain, but *Lactobacillus delbrueckii *has two genes encoding dsRHbd; one is associated with the RNase HI domain and the other is associated with the resolvase domain. In addition, it is interesting that the dsRHbds of *Gloeobacter violaceus*, *Bdellovibrio bacteriovorus*, and *Myxococcus xanthus *were identified in the C-terminus of RNase HI even though many dsRHbds were in the N-terminus, as in the eukaryotes. Multiple alignments of the amino acid sequences of prokaryotic dsRHbds showed that the sequences of dsRHbd located in the C-terminus were similar (Figure 4). The process of dsRHbd acquisition can be inferred from the fact that almost half of the RNase HI with dsRHbd was found in firmicutes that have the abilities to acquire new genes through lateral gene transfer [36]. In addition, RNase HIII genes were not found in any genomes of the 31 species that encoded RNase HI with dsRHbd (Additional file 3), supporting the hypothesis of mutually exclusive evolution of RNase HI and HIII.

**Table 3:** List of genes containing dsRHbd in complete genomes.

Kingdom	Species	Accession No.	ORF	Direction	Domain
Archaea	**Euryarchaeota**				
	*Methanococcus maripaludis*	NC_005791	832383–832988	complement	dsRHbd (4–46), RNH (65–201)
					
Bacteria	**Bacteroidetes**				
	*Bacteroides fragilis *NCTC 9343	NC_003228	207338–207967	complement	dsRHbd (6–48), RNH (79–197)
	*Bacteroides fragilis *YCH46	NC_006347	253987–254616	complement	dsRHbd (6–48), RNH (79–197)
	*Bacteroides thetaiotaomicron*	NC_004663	4371820–4372455	complement	dsRHbd (6–48), RNH (81–199)
	*Porphyromonas gingivalis*	NC_002950	1292573–1293223	direct	dsRHbd (5–47), RNH (85–199)
					
	**Cyanobacteria**				
	*Gloeobacter violaceus*	NC_005125	3808044–3808685	complement	RNH (1–138), dsRHbd (156–197)
					
	**Firmicutes**				
	*Bacillus clausii*	NC_006582	1399495–1400094	direct	dsRHbd (6–48), RNH (74–183)
	*Bacillus halodurans*	NC_002570	933504–934094	direct	dsRHbd (6–48), RNH (69–192)
	*Clostridium acetobutylicum*	NC_003030	2659515–2660237	complement	dsRHbd (6–48), RNH (103–238)
	*Clostridium perfringens*	NC_003366	1707913–1708542	complement	dsRHbd (5–49), RNH (70–207)
	*Clostridium tetani*	NC_004557	2281472–2282092	complement	dsRHbd (5–47), RNH (68–203)
	*Desulfitobacterium hafniense*	NC_007907	2075150–2075770	complement	dsRHbd (8–50), RNH (71–203)
	*Lactobacillus acidophilus*	NC_006814	116459–117205	direct	dsRHbd (3–45), RNH (91–245)
	*Lactobacillus delbrueckii*	NC_008054	146497–147264	direct	dsRHbd (4–45), RNH (93–253)
			978609–979283	complement	Resolvase (1–106), dsRHbd (127–168)
	*Lactobacillus johnsonii*	NC_005362	118550–119281	direct	dsRHbd (3–45), RNH (86–240)
	*Lactobacillus plantarum*	NC_004567	2310574–2311470	direct	dsRHbd (5–47), RNH (70–225)
	*Lactobacillus salivarius*	NC_007929	459722–460381	direct	dsRHbd (4–46), RNH (59–216)
	*Mesoplasma florum*	NC_006055	559864–560484	complement	dsRHbd (3–45), RNH (63–199)
	*Mycoplasma capricolum*	NC_007633	382602–383222	direct	dsRHbd (5–47), RNH (63–198)
	*Mycoplasma mycoides*	NC_005364	375791–376408	direct	dsRHbd (5–47), RNH (62–198)
	*Mycoplasma penetrans*	NC_004432	1267458–1268120	complement	dsRHbd (5–47), RNH (73–216)
					
	**Fusobacteria**				
	*Fusobacterium nucleatum*	NC_003454	1651474–1652124	complement	dsRHbd (6–48), RNH (70–215)
					
	**Deltaproteobacteria**				
	*Bdellovibrio bacteriovorus*	NC_005363	3036447–3037238	complement	RNH (18–164), dsRHbd (207–248)
	*Desulfotalea psychrophila*	NC_006138	1023218–1024003	direct	dsRHbd (28–70), RNH (105–247)
	*Myxococcus xanthus*	NC_008095	2628106–2628873	complement	RNH (3–154), dsRHbd (199–240)
					
	**Gammaproteobacteria**				
	*Colwellia psychrerythraea*	NC_003910	1743847–1744665	complement	dsRHbd (5–47), RNH (98–247)
	*Photobacterium profundum*	NC_006370	2161121–2161870	complement	dsRHbd (5–47), RNH (77–225)
	*Saccharophagus degradans*	NC_007912	82187–82945	complement	dsRHbd (5–47), RNH (81–228)
	*Shewanella denitrificans*	NC_007954	880428–881219	direct	dsRHbd (5–47), RNH (91–239)
					
	**Spirochaetes**				
	*Borrelia burgdorferi*	NC_001318	897096–897740	complement	dsRHbd (19–62), RNH (74–211)
	*Borrelia garinii*	NC_006156	899069–899668	complement	dsRHbd (4–47), RNH (88–196)
					
	**Thermotogae**				
	*Thermotoga maritima*	NC_000853	1322788–1323459	complement	dsRHbd (7–49), RNH (63–197)

dsRHbd sequences were found in 32 complete genomes (31 species, one with two strains). Open reading frame (ORF) numbers indicate the genomic positions of the genes that encode dsRHbd. Domain numbers indicate the amino acid positions relative to the start of each protein sequence. RNH represents the RNase H group.

### Redundant RNase HI genes in a single genome

We also found that 10 of the 31 species listed in Table 3 had multiple RNase HI genes (see Additional file 3). If RNase HI with a dsRHbd gene influences the existence of the RNase HIII gene in a genome, how is the effect exerted on other RNase HI genes? To address this question, we examined the amino acid sequences of RNase HI without dsRHbd in these 10 species. The RNase HI without dsRHbd that were found in five species in the firmicutes and one species in the deltaproteobacteria, with the exception of *B. bacteriovorus*, were similar in structure (e.g., lacked the basic protrusion) to that of the Group A RNase HI (see Figure 3). On the other hand, the primary structures of RNase HI without dsRHbd in three species of gammaproteobacteria resembled that of *E. coli*, and there were few differences in their amino acid sequences. Because the primary structures of RNase HI with dsRHbd in the same species in the gammaproteobacteria were also similar to that of *E. coli*, it is difficult to distinguish redundant RNase HI genes on the basis of their amino acid similarities.

**Figure 5:**
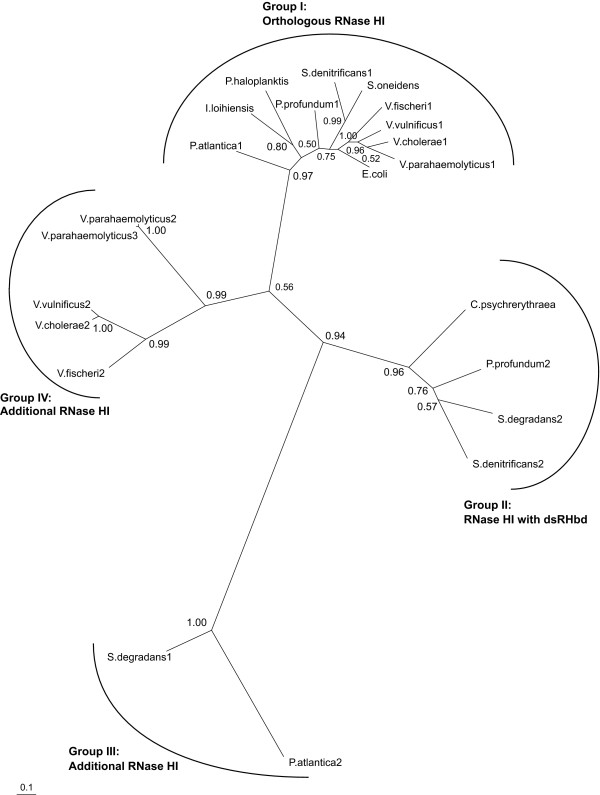
**Bayesian phylogenetic tree for the RNase HI domains**. A phylogenetic tree was constructed by using Bayesian inference on the basis of the alignments of RNase HI domain sequences from 12 species in the gammaproteobacteria, as listed in Additional File 4. Species names followed by Arabic numerals are used to distinguish multiple forms of RNase HI in a genome; names not followed by Arabic numerals indicate the presence of a single RNase HI in a genome. Numbers at the nodes represent posterior probabilities. The scale bar equals 0.1 substitutions per site.

To identify the differences among redundant RNase HI sequences of the gammaproteobacteria (see Additional file 4), we constructed a Bayesian tree based on the nucleotide sequences of the RNase HI domains from 12 species in the gammaproteobacteria (Figure 5). This analysis divided the RNase HI domains into four gene clusters: orthologous RNase HI, including *E. coli *RNase HI (Group I); RNase HI with dsRHbd (Group II); and other two groups of additional RNase HI (Groups III and IV). Because RNase HI genes in Group I appear to have been inherited by vertical descent from a common ancestor, we defined them as orthologous RNase HI genes. On the other hand, RNase HI genes of Group II to IV seem to have been provided by gene duplication or lateral gene transfer in addition to the original RNase HI genes. Interestingly, orthologous RNase HI was not found in *Saccharophagus degradans *that contains RNase HI with dsRHbd (Group II). In contrast, *Pseudoalteromonas atlantica *contains orthologous RNase HI (Group I) instead of RNase HI with dsRHbd, though the presence of Group III RNase HI is common to *S. degradans *and *P. atlantica*. In addition, orthologous RNase HI was not found in the genome of *Colwellia psychrerythraea*, which contains only RNase HI with a dsRHbd gene (Group II). The same statement applies to 21 other prokaryotic species that have only RNase HI with dsRHbd (see Additional file 3). On the other hand, we also found that orthologous RNase HI (Group I) and RNase HI with dsRHbd (Group II) had both been retained in two genomes of *Photobacterium profundum *and *Shewanella denitrificans*. These results suggest that RNase HI with dsRHbd may be capable of replacing the original RNase HI. A lineage-specific characterization such as the mapping of gene trees onto species trees using a soft parsimony algorithm [37] is necessary for more precise analysis of the transition of RNase HI genes during the course of evolution.

## Discussion

Using genome-wide and phylogenetic analyses of RNase H genes, we obtained the following findings: (1) most bacterial species had combinations of RNase HI and HII (80%, 189 out of 235 species) or RNase HII and HIII (7%, 16 out of 235 species); (2) the combination of RNase HI and HIII genes was not found in any species (0% in Group D) unless RNase HII was also present (Group A; 15 species in the firmicutes); (3) the combination of RNase H genes has been altered, even in closely related species, in such a way that the functional RNase HI and HIII genes do not coexist in a single genome; (4) dsRHbd was found in RNase HI in 31 out of 189 species (16%) that contain the RNase HI and HII genes; (5) dsRHbd was not found in the RNase HI in all 15 species that contained all three types of RNase H genes; and (6) RNase HI with dsRHbd may have replaced the orthologous RNase HI without dsRHbd in 21 out of 31 species (68%) that have RNase HI with dsRHbd.

To ascertain the cause of the mutually exclusive evolution of RNase HI and RNase HIII, we focused on their enzymatic properties. Previous reports have indicated that RNase HI and HIII digest the RNA moiety of RNA-DNA hybrids such as Okazaki fragments more effectively than is the case for RNase HII [38], whereas only RNase HII is capable of removing a single ribonucleotide of DNA-RNA-DNA/DNA hybrids such as an RNA that has been misincorporated into DNA [15,16]. In addition, mutagenesis analyses of *B. subtilis *RNase H genes have shown that single-gene knockout mutants targeting the RNase HII or HIII genes exhibit normal growth, but that double-knockout mutants for both genes are unable to form viable colonies; this suggests that a functional overlap exists between RNase HII and HIII [31]. On the other hand, the existence of functional redundancy between RNase HI and HII is not clear, although double-knockout mutants of *E. coli *RNase HI and HII exhibit a temperature-sensitive phenotype (Dr. Mitsuhiro Itaya, Keio University, personal communication). We hypothesize that the functional similarities and differences among the three RNase H genes may explain this evolutionary process, because a theoretical model of genetic redundancy suggests that the fates of redundant genes are likely to depend on the extent of their functional redundancy [5].

According to computer simulations using a genetic redundancy model [5], redundant genes do not persist when they are equally effective at performing their functions (Model 1). On the other hand, redundant genes are evolutionarily stable in two situations: when both genes perform the same function but one is less efficient than the other gene (Model 2), and when the main functions of the two genes differ but one of the genes functions similarly to the other gene, but with lower efficiency (Model 3). The insights from this simulation can be applied to the molecular evolution of RNase H genes in prokaryotes. At first glance, the reason why most bacterial species have combinations of RNase HI with RNase HII or RNase HII with RNase HIII can be explained by Model 3; that is, these combinations are evolutionarily stable because both genes in each combination have independent functions but with an unknown degree of functional overlap. Likewise, the combination of RNase HI and HIII genes is evolutionarily unstable owing to their functional redundancy (Model 1), and this may explain why no species has both functional genes in its genome and why the combination of RNase H genes has been altered even in closely related species in such a way that RNase HI and HIII genes will not coexist in a single genome.

**Figure 6:**
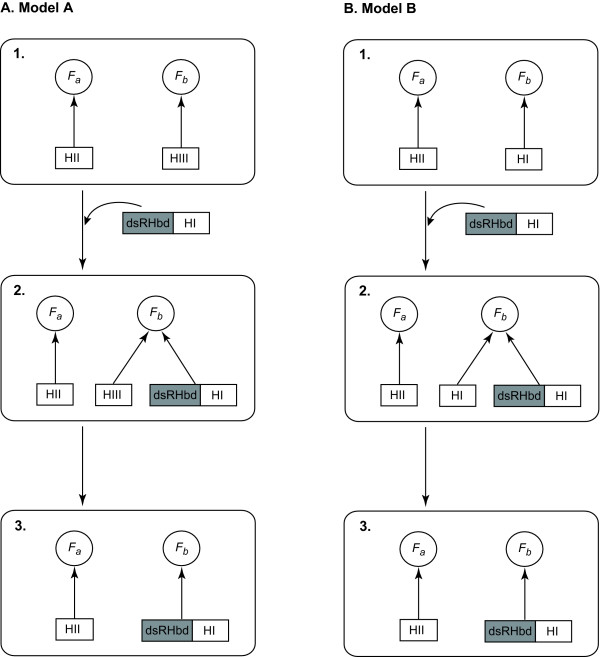
**Possible evolutionary models for the three RNase H genes**. Two models that can explain the mutually exclusive evolution of RNase HIII and RNase HI with dsRHbd (Model A) and of RNase HI with and without dsRHbd (Model B). *F*_*a *_and *F*_*b *_indicate the different functions of the RNase H enzymes.

It seems that the effect of functional redundancy is more severe for RNase HI with dsRHbd in firmicutes, because RNase HIII was found in all 15 species whose genomes encode RNase HI without dsRHbd but was not found in any of the 31 species containing RNase HI with dsRHbd. Given the distribution pattern of RNase HI with dsRHbd in prokaryotes, we proposed the following process: once RNase HI with dsRHbd is acquired (for example, by lateral gene transfer [39]), the combination of RNase HI with dsRHbd and RNase HIII may become evolutionarily unstable owing to their functional redundancy, and one of them is subsequently removed during the course of evolution. We propose this evolutionary process as Model A in Figure 6A. In particular, it is interesting that the RNase H combinations of two species that are regarded as the deepest branching organisms are different: *Thermotoga maritima *has a combination of RNase HI with dsRHbd and RNase HII, whereas *Aquifex aeolicus *retains a combination of RNase HII and HIII [31]. This may reflect the ancient status of these RNase H combinations in bacteria, and RNase HIII might have been altered along with RNase HI with dsRHbd in *T. maritima *in accordance with our model. Also, the fact that RNase HIII genes are less abundant than those of RNase HI and HII (Table 1) suggests the possibility that RNase HIII genes have been replaced in genomes by other RNase H genes during the course of evolution.

A similar scenario might have occurred in the case of redundant RNase HI genes, because our findings also suggest that RNase HI with dsRHbd may have replaced the existing RNase HI without dsRHbd in 21 species. As shown in Model B (Figure 6B), once RNase HI with dsRHbd is obtained, it competes with RNase HI without dsRHbd because of their functional redundancy, and one of them is excluded. It is also noteworthy that RNase HI with dsRHbd is encoded as a single-copy gene in prokaryotic genomes (Table 3). Interestingly, human genome contains one RNase H1 with dsRHbd and at least three pseudogenes related to RNase H1 with dsRHbd [40]. Although we previously showed that four RNase H1-encoding genes in *Caenorhabditis elegans *exhibited gene-specific expression patterns during development; one gene encodes RNase H1 with dsRHbd and other three gene encode RNase H1 without dsRHbd [41], it was also found that most of the eukaryotic genomes contained single-copy genes encoding RNase H1 with dsRHbd but RNase H1 without dsRHbd had rarely been identified in the same genome (data not shown). Functional characteristic of RNase H1 with dsRHbd seems to depend on eukaryotic species because disruptions of RNase H1 with dsRHbd resulted in lethal phenotype of fly [42] and mice [13] but showed normal growth in yeast [43] and trypanosoma [44]. However, it appears that prokaryotic and eukaryotic genomes have single-copy RNase H1 with dsRHbd and this tendency might be explained by functional redundancy within individual genomes. In a future work, more detailed analysis of eukaryotic RNase H1 genes is required to show the effect of functional redundancy on the evolution of redundant genes in eukaryotes.

We also discovered that several RNase HI genes could exist in a single genome (Table 1) and that there are some cases in which orthologous RNase HI is retained in the presence of RNase HI with dsRHbd (Figure 5). This raises the question of how multiple RNase HI genes can be retained in a single genome. This is difficult to explain using the genetic evolution models described in Figure 6, because multiple RNase HI genes should also have the same function and should be subject to the same mechanisms that govern the fate of redundant genes. In the case of duplicated genes, the usual fate of redundant genes is that one is silenced through a strong purifying selection after a brief period of relaxed selection [45]. The number of RNase HI genes differed among species even within the same lineages, suggesting that gene duplication or gene transfer might have occurred relatively recently and that redundant genes may have arisen during a period of relaxed selection. An alternative possible explanation for multiple RNase HI is that neofunctionalization and subfunctionalization have been shown by computer simulation to increase the retention rate of duplicated genes [46,47]. Although it is not known whether the retention of multiple RNase HI genes resulted from subfunctionalization or neofunctionalization, RNase HI appears to represent the acquisition of a new function based on the example of *Streptomyces coelicolor *A3(2), which encodes a bifunctional enzyme consisting of an RNase H domain and an acid phosphatase domain [48]. In addition to subfunctionalization, it is also possible that some of the RNase HI genes identified in this study might be in the process of nonfunctionalization (pseudogenization) and can be expected to become pseudogenes [49]. Actually, genomic sequences encoding truncated RNase HI domains have been found in some species during genome-wide identification of RNase H genes (data not shown), suggesting the existence of one or more nonfunctionalized RNase HI gene in our dataset. Moreover, even if the coding sequences seem not to have been nonfunctionalized, the regulatory regions might have mutations because duplicated genes are considered to be under active selection pressure owing to energy constraints on gene expression [50]. Further investigations will be necessary to reveal the effect of each functionalization on multiple RNase HI genes in prokaryotes.

In this study, two possible models were provided to explain the evolution of RNase H combinations in prokaryotic genomes. We believe that our models are the first example of the effects of functional redundancy on changes in gene constitution during the course of gene evolution. Experimental evolution of bacterial species constructed to have mutually exclusive genes by means of genetic engineering may be effective in verification of our models. For example, RNase HI and RNase HIII genes tagged with different drug resistances are inserted into the RNase HI-knockout mutants of *E. coli *and repeated subcultures of the recombinants allow us to detect the mutated RNase H gene using specific drug resistances as markers. This experimental approach would certainly be worthwhile to explore the fate of redundant RNase H genes in future research.

## Conclusion

We identified three genes that encode RNase H enzymes and examined the combinations of these genes in 353 prokaryotic genomes. Our results showed that RNase H combinations might have evolved in such a way that the RNase HI and HIII genes will not be inherited together within an individual genome and that this tendency is prominent when RNase HI contains dsRHbd. This mutually exclusive evolution of RNase H genes seems to be related to functional redundancy, because previous reports have suggested that the substrate preferences of RNase HI and HIII are similar. Taken together, these results suggest possible evolutionary models for the RNase H genes in which functional redundancy contributes to the exclusion of redundant genes. Our findings thus provide a good example of the effects of functional redundancy on gene evolution, confirming certain theoretical predictions.

## Methods

### Genome-wide identification of genes encoding RNase H and dsRHbd

Complete genomes of 326 strains from 235 bacterial species and 27 strains from 27 archaeal species and the corresponding GenBank files were downloaded from the National Center for Biotechnology Information (NCBI) GenBank FTP site [51]; their accession numbers are summarized in Additional file 1. Two strategies were applied to identify sequences of RNase H and double-stranded RNA and RNA-DNA hybrid-binding domains (dsRHbd) in the complete genomes. One was a remote homology search with the PSI-BLAST software [52] and the other was a protein domain search based on Hidden Markov Model (HMM) profiles [53].

For the PSI-BLAST search, a non-redundant peptide sequence database was downloaded from the NCBI BLAST FTP site [54]. From this database, peptide sequences of prokaryotes and eukaryotes were extracted by using taxonomy information obtained from the NCBI Taxonomy FTP site [55]. To construct a position-specific scoring matrix, a PGP-BLAST search was carried out against 3 506 454 extracted peptide sequences, with an *E*-value threshold of 0.002 and four iterations. The amino acid and nucleotide sequences corresponding to the RNase HI domain of *E. coli *K12 [GenBank: AAC73319], the RNase HII domain of *E. coli *K12 [GenBank: AAC73294], the RNase HIII domain of *B. subtilis *subsp. subtilis str. 168 [Swissprot: P94541], and the dsRHbd of *B. halodurans *C-125 [Swissprot: Q9KEI9] were used as queries. Using the resulting matrix, PSI-TBLASTN searches were conducted against the 353 complete genomes by using an *E*-value threshold of 0.2.

For the HMM profile analysis, the profiles of RNase HI and RNase HII were downloaded from the Sanger Institute's Pfam Web site [56] and the HMM profile of dsRHbd was newly built by using the hmmbuild module of the HMMER 2.3.2 software [53] on the basis of the results of the PSI-BLAST search. The 353 complete genomes were translated into six-frame amino acid sequences. Using these HMM profiles as queries, protein domain searches were performed with the hmmpfam module of the HMMER 2.3.2 software against translated complete genomes with an *E*-value threshold of 1× 10^-6^.

On the basis of the outputs of the PSI-BLAST and HMM searches, coding sequences including homologous regions of RNase H or dsRHbd were obtained from GenBank files by using G-language Perl modules [57]. When the search revealed unannotated genomic regions, we manually checked for the existence of an open reading frame (ORF) near the genomic region. In order to distinguish genes encoding RNase HII and RNase HIII in the datasets, a PGPBLAST search was conducted against the Conserved Domain Database (a subset of domains from SMART, Pfam, COG, and CD) [58] downloaded from the NCBI CDD FTP site [59].

### Phylogenetic analysis

The amino acid and nucleotide sequences of the DNA gyrase subunit B gene (*gyrB*) were retrieved in a similar way. The CodonAlign 2.0 software (Barry G. Hall, Rochester, NY, USA) was used to align the nucleotide sequences on the basis of alignments of the corresponding amino acid sequences performed with the ClustalW 1.8.3 software [60]. The Modeltest 3.7 software [61] was applied to select an appropriate model from the output of the PAUP* Version 4.0 software [62] by using hierarchical likelihood-ratio tests and the Akaike Information Criterion [63]. Phylogenetic trees were estimated by Bayesian methods with MRBAYES Version 3.1.2 software [64] under the General Time Reversible model with gamma correction and a proportion of invariable sites [65]. In the Bayesian analysis, the Markov chain Monte Carlo search used 1 000 000 generations run with four chains, with trees being sampled every 100 generations, and a consensus tree was estimated by a burn-in of 2500 trees. TreeView software for Power Macintosh [66] was used for viewing and editing the tree.

## Abbreviations

RNase H, ribonuclease H; dsRHbd, double-stranded RNA and RNA-DNA hybrid-binding domains; dsRNA, double-stranded RNA; *gyrB*, DNA gyrase subunit B gene; HMM, Hidden Markov Model; ORF, open reading frame.

## Authors' contributions

HK conceived the study. MT and AK supervised this work. All authors read and approved the final version of the manuscript.

## Supplementary Material

Additional file 1 List of genes containing the RNase H domain from 27 archaea and 326 bacteria. Click here for file

Additional file 2 RNase HI sequences of the bacillales and lactobacillales used for the multiple alignments. Click here for file

Additional file 3 List of RNase H genes from the species whose genomes encode dsRHbd sequences. Click here for file

Additional file 4 RNase HI sequences from 12 species in the gammaproteobacteria used for the Bayesian phylogenetic analysis. Click here for file
