# Supplementary material for: Evolution of ribonuclease H genes in prokaryotes to avoid inheritance of redundant genes
Source: BMC Evol Biol. 2007 Jul 31;7:128. doi: 10.1186/1471-2148-7-128 (PMC1950709; doi:10.1186/1471-2148-7-128)
Supplement: Additional file 1 — List of genes containing the RNase H domain from 27 archaea and 326 bacteria. [file 1471-2148-7-128-S1.pdf]

Additional file 1: List of genes containing RNase H domain from A) archaea and B) bacteria. ORF numbers indicate the genomic positions of the genes encoding RNase H. Domain numbers indicate the amino acid positions relative to the start of each protein sequences. The RNase H combination refers to the groups defined in Figure 1. Apostrophes (i.e., B') represent the presence of dsRHbd.

A) List of RNase H genes in archaea

Species	Type	Accession No.	ORF	Direction	Domain	Combination
**Crenarchaeota**						
Aeropyrum pernix K1	RNase HII	NC 000854	344881-345564	direct	7-203	F
Pyrobaculum aerophilum str. IM2	RNase HI	NC 003364	1054486-1055061	complement	1-155	B
	RNase HII	NC_003364	715015-715653	complement	3-190	
Sulfolobus acidocaldarius DSM 639	RNase HI	NC 007181	1479253-1479699	direct	1-148	B
	RNase HII	NC_007181	766641-767279	complement	4-197	
Sulfolobus solfataricus P2	RNase HII	NC 002754	2170481-2171119	direct	3-195	F
Sulfolobus tokodaii str. 7	RNase HI	NC 003106	733569-734018	complement	1-149	B
	RNase HII	NC_003106	514734-515360	direct	4-196	
**Euryarchaeota**						
Archaeoglobus fulgidus DSM 4304	RNase HII	NC 000917	558053-558670	complement	3-194	F
Haloarcula marismortui ATCC 43049	RNase HI	NC 006396	2936421-2937017	direct	66-198	B
	RNase HI	NC_006396	1565617-1566279	complement	31-176	
	RNase HII	NC_006396	2842265-2842900	complement	3-196	
Halobacterium sp. NRC-1	RNase HI	NC 002607	209439-210038	direct	66-199	B
	RNase HI	NC_002607	1211764-1212387	complement	31-174	
	RNase HII	NC_002607	1464785-1465423	direct	4-197	
Methanocaldococcus jannaschii DSM 2661	RNase HII	NC 000909	130417-131109	complement	4-215	F
Methanococcoides burtonii DSM 6242	RNase HII	NC 007955	443348-443989	direct	4-208	F
Methanococcus maripaludis S2	RNase HI'	NC 005791	832383-832988	complement	65-201	B'
	RNase HII	NC_005791	1355645-1356373	direct	24-225	
Methanopyrus kandleri AV19	RNase HII	NC 003551	178822-179454	direct	5-194	F
Methanosarcina acetivorans C2A	RNase HI	NC 003552	744362-745201	direct	3-145	B
	RNase HII	NC_003552	2423163-2423834	direct	4-210	
Methanosarcina barkeri str. fusaro	RNase HII	NC 007355	3500465-3501139	direct	4-210	F
Methanosarcina mazei Go1	RNase HII	NC 003901	3377458-3378120	direct	4-210	F
Methanosphaera stadtmanae DSM 3091	RNase HII	NC 007681	1564036-1564674	direct	10-204	C
	RNase HIII	NC_007681	643872-644801	direct	113-305	
Methanospirillum hungatei JF-1	RNase HI	NC 007796	1658736-1659140	direct	2-134	B
	RNase HII	NC_007796	1384014-1384652	complement	3-197	
Methanothermobacter thermautotrophicus str. Delta H	RNase HII	NC 000916	935622-936242	complement	4-201	F
Natronomonas pharaonis DSM 2160	RNase HI	NC 007426	2517025-2517612	direct	66-195	B
	RNase HI	NC_007426	859881-860543	complement	30-176	
	RNase HII	NC_007426	1158901-1159539	direct	5-197	
Picrophilus torridus DSM 9790	RNase HII	NC 005877	1271360-1271959	direct	4-191	F
Pyrococcus abyssi GE5	RNase HII	NC 000868	495426-496100	direct	4-201	F
Pyrococcus furiosus DSM 3638	RNase HII	NC 003413	1655335-1656009	direct	4-201	F
Pyrococcus horikoshii OT3	RNase HII	NC 000961	1456852-1457514	complement	4-201	F
Thermococcus kodakarensis KOD1	RNase HII	NC 006624	703189-703875	direct	4-201	F
Thermoplasma acidophilum DSM 1728	RNase HII	NC 002578	1504217-1504855	complement	8-198	F
Thermoplasma volcanium GSS1	RNase HII	NC 002689	142583-143242	complement	14-204	F
**Nanoarchaeota**						
Nanoarchaeum equitans Kin4-M	RNase HII	NC 005213	58428-59030	complement	4-194	F

B) List of RNase H genes in bacteria

Species	Type	Accession No.	ORF	Direction	Domain	Combination
**Acidobacteria**						
Acidobacteria bacterium Ellin345	RNase HI	NC_008009	89263-89898	complement	14-164	B
	RNase HII	NC_008009	3420785-3421549	direct	49-225	
**Actinobacteria**						
Bifidobacterium longum NCC2705	RNase HI	NC_004307	2031348-2032334	complement	32-185	B
	RNase HII	NC_004307	1197464-1198303	complement	21-279	
Corynebacterium diphtheriae NCTC 13129	RNase HI	NC_002935	1715369-1716502	complement	5-151	B
	RNase HII	NC_002935	1538491-1539180	complement	37-216	
Corynebacterium efficiens YS-314	RNase HI	NC_004369	2267409-2268716	complement	49-196	B
	RNase HII	NC_004369	2032763-2033458	complement	26-205	
Corynebacterium glutamicum ATCC 13032 Bielefeld	RNase HI	NC_006958	2343082-2344230	complement	5-152	B
	RNase HI	NC_006958	341348-342379	direct	180-318	
	RNase HII	NC_006958	2116613-2117281	complement	23-202	
Corynebacterium glutamicum ATCC 13032 Kitasato	RNase HI	NC_003450	2371410-2372558	complement	5-152	B
	RNase HI	NC_003450	341356-342378	direct	177-315	
	RNase HII	NC_003450	2146563-2147231	complement	23-202	
Corynebacterium jeikeium K411	RNase HI	NC_007164	804931-806226	direct	3-157	B
	RNase HII	NC_007164	1381137-1381763	complement	14-194	
Frankia sp. CcI3	RNase HI	NC_007777	1731165-1732301	direct	9-156	B
	RNase HII	NC_007777	4287805-4288527	complement	30-209	
Leifsonia xyli subsp. xyli str. CTCB07	RNase HI	NC_006087	1570484-1571362	direct	4-138	B
	RNase HII	NC_006087	1541158-1541838	complement	25-212	
Mycobacterium avium subsp. paratuberculosis K-10	RNase HI	NC_002944	2186416-2187549	complement	5-151	B
	RNase HII	NC_002944	3305131-3305850	complement	33-212	
Mycobacterium bovis AF2122/97	RNase HI	NC_002945	2480792-2481886	complement	5-151	B
	RNase HII	NC_002945	3168678-3169472	complement	36-215	
Mycobacterium leprae TN	RNase HI	NC_002677	1974010-1975125	complement	5-151	B
	RNase HII	NC_002677	1935438-1936160	complement	36-213	
Mycobacterium sp. MCS	RNase HI	NC_008146	3542028-3543125	complement	5-151	B
	RNase HI	NC_008146	4628235-4628699	direct	3-144	
	RNase HII	NC_008146	2119111-2119830	direct	33-212	
Mycobacterium tuberculosis CDC1551	RNase HI	NC_002755	2498919-2500013	complement	5-151	B
	RNase HII	NC_002755	3206316-3207110	complement	36-215	
Mycobacterium tuberculosis H37Rv	RNase HI	NC_000962	2501644-2502738	complement	5-151	B
	RNase HII	NC_000962	3212162-3212956	complement	36-215	
Nocardia farcinica IFM 10152	RNase HI	NC_006361	5964453-5964998	direct	2-140	B
	RNase HI	NC_006361	1789965-1791185	direct	3-154	
	RNase HI	NC_006361	4164152-4164802	complement	30-179	
	RNase HII	NC_006361	4291410-4292165	complement	35-214	
Propionibacterium acnes KPA171202	RNase HI	NC_006085	1885186-1886088	complement	3-137	B
	RNase HII	NC_006085	1551333-1551947	complement	16-195	
Rubrobacter xylanophilus DSM 9941	RNase HII	NC_008148	1416531-1417145	direct	28-202	C
	RNase HIII	NC_008148	831365-832300	direct	92-294	
Streptomyces avermitilis MA-4680	RNase HI	NC_003155	7086857-7088173	direct	7-153	B
	RNase HI	NC_003155	1426227-1426937	complement	6-150	
	RNase HII	NC_003155	2991649-2992350	complement	24-202	
Streptomyces coelicolor A3(2)	RNase HI	NC_003888	8089935-8090642	direct	7-150	B
	RNase HI	NC_003888	2468071-2469564	complement	14-160	
	RNase HII	NC_003888	6356449-6357150	direct	24-202	
Symbiobacterium thermophilum IAM 14863	RNase HI	NC_006177	3150382-3150825	complement	1-142	B
	RNase HII	NC_006177	1612404-1613183	direct	71-248	
	RNase HII	NC_006177	2171084-2171719	complement	18-205	
Thermobifida fusca YX	RNase HI	NC_007333	2284461-2285609	complement	11-158	B
	RNase HII	NC_007333	2513701-2514396	complement	28-206	
Tropheryma whipplei str. Twist	RNase HI	NC_004572	322955-323440	complement	3-141	E
Tropheryma whipplei TW08/27	RNase HI	NC_004551	569929-570414	direct	3-141	E
**Aquificae**						
Aquifex aeolicus VF5	RNase HII	NC_000918	1374521-1375111	direct	16-191	C
	RNase HIII	NC_000918	1245528-1246301	direct	74-257	
**Bacteroidetes**						
Bacteroides fragilis NCTC 9343	RNase HI'	NC_003228	207338-207967	complement	79-209	B'
	RNase HII	NC_003228	263354-263959	direct	13-191	
Bacteroides fragilis YCH46	RNase HI'	NC_006347	253987-254616	complement	79-209	B'
	RNase HII	NC_006347	318671-319276	direct	13-191	
Bacteroides thetaiotaomicron VPI-5482	RNase HI'	NC_004663	4371820-4372455	complement	81-211	B'
	RNase HII	NC_004663	4395851-4396453	direct	13-191	
Porphyromonas gingivalis W83	RNase HI'	NC_002950	1292573-1293223	direct	85-216	B'
	RNase HII	NC_002950	785748-786353	direct	14-192	
Salinibacter ruber DSM 13855	RNase HI	NC_007677	1397735-1398658	complement	166-294	B
	RNase HI	NC_007677	1496687-1497271	complement	47-191	
	RNase HII	NC_007677	2949482-2950084	complement	17-198	

B) List of RNase H genes in bacteria (Continued)

Species	Type	Accession No.	ORF	Direction	Domain	Combination
**Chlamydiae**						
Candidatus Protochlamydia amoebophila UWE25	RNase HII	NC_005861	803632-804300	direct	32-210	C
	RNase HIII	NC_005861	827796-828701	complement	93-292	
Chlamydia muridarum Nigg	RNase HII	NC_002620	355302-355955	direct	30-207	C
	RNase HIII	NC_002620	330423-331328	complement	91-293	
Chlamydia trachomatis A/HAR-13	RNase HII	NC_007429	33750-34403	direct	30-207	C
	RNase HIII	NC_007429	8812-9714	complement	89-291	
Chlamydia trachomatis D/UW-3/CX	RNase HII	NC_000117	33154-33807	direct	30-207	C
	RNase HIII	NC_000117	8217-9119	complement	89-291	
Chlamydophila abortus S26/3	RNase HII	NC_004552	717265-717903	complement	31-208	C
	RNase HIII	NC_004552	338573-339475	complement	89-291	
Chlamydophila caviae GPIC	RNase HII	NC_003361	747070-747708	complement	31-208	C
	RNase HIII	NC_003361	341067-341969	complement	89-291	
Chlamydophila felis Fe/C-56	RNase HII	NC_007899	419621-420250	direct	29-206	C
	RNase HIII	NC_007899	819694-820596	direct	89-291	
Chlamydophila pneumoniae AR39	RNase HII	NC_002179	690034-690678	complement	30-207	C
	RNase HIII	NC_002179	848165-849070	direct	92-294	
Chlamydophila pneumoniae CWL029	RNase HII	NC_000922	150523-151167	direct	30-207	C
	RNase HIII	NC_000922	1222362-1223258	complement	89-291	
Chlamydophila pneumoniae J138	RNase HII	NC_002491	150234-150878	direct	30-207	C
	RNase HIII	NC_002491	1218696-1219592	complement	89-291	
Chlamydophila pneumoniae TW-183	RNase HII	NC_005043	148018-148662	direct	30-207	C
	RNase HIII	NC_005043	1218066-1218962	complement	89-291	
**Chlorobi**						
Chlorobium chlorochromatii CaD3	RNase HI	NC_007514	2262878-2263318	direct	2-143	B
	RNase HII	NC_007514	2530005-2530625	complement	17-200	
Chlorobium tepidum TLS	RNase HI	NC_002932	1518500-1518940	direct	2-143	B
	RNase HII	NC_002932	2125957-2126574	direct	18-200	
Pelodictyon luteolum DSM 273	RNase HI	NC_007512	1799552-1800013	direct	9-150	B
	RNase HII	NC_007512	15317-15943	complement	20-203	
**Chloroflexi**						
Dehalococcoides ethenogenes 195	RNase HI	NC_002936	342416-342820	direct	1-134	B
	RNase HII	NC_002936	717797-718432	direct	24-204	
Dehalococcoides sp. CBDB1	RNase HI	NC_007356	262411-262815	direct	1-134	B
	RNase HII	NC_007356	617419-618057	direct	25-205	
**Cyanobacteria**						
Anabaena variabilis ATCC 29413	RNase HI	NC_007413	1859789-1860697	direct	5-148	B
	RNase HII	NC_007413	1572821-1573498	direct	38-220	
Gloeobacter violaceus PCC 7421	RNase HI'	NC_005125	3808044-3808685	complement	1-138	B'
	RNase HII	NC_005125	1622346-1622942	direct	2-178	
Nostoc sp. PCC 7120	RNase HI	NC_003272	147532-148440	direct	5-148	B
	RNase HII	NC_003272	5186375-5187052	direct	38-220	
Prochlorococcus marinus str. MIT 9312	RNase HI	NC_007577	194846-195565	complement	9-148	B
	RNase HII	NC_007577	1492848-1493465	direct	19-201	
Prochlorococcus marinus str. MIT 9313	RNase HI	NC_005071	2205905-2206678	direct	6-149	B
	RNase HII	NC_005071	1886298-1886954	complement	27-209	
Prochlorococcus marinus str. NATL2A	RNase HI	NC_007335	1531032-1531517	complement	6-149	B
	RNase HII	NC_007335	1009741-1010328	direct	9-191	
Prochlorococcus marinus subsp. marinus str. CCMP1375	RNase HI	NC_005042	224331-224828	complement	6-149	B
	RNase HII	NC_005042	1520802-1521377	direct	7-189	
Prochlorococcus marinus subsp. pastoris str. CCMP1986	RNase HI	NC_005072	194249-194971	complement	6-149	B
	RNase HII	NC_005072	1442320-1442949	direct	23-205	
Synechococcus elongatus PCC 6301	RNase HI	NC_006576	2054689-2055474	direct	5-148	B
	RNase HII	NC_006576	733973-734620	complement	22-204	
Synechococcus elongatus PCC 7942	RNase HI	NC_007604	2261556-2262335	complement	3-146	B
	RNase HII	NC_007604	886245-886892	direct	22-204	
Synechococcus sp. CC9605	RNase HI	NC_007516	2272572-2273330	complement	6-149	B
	RNase HII	NC_007516	320086-320685	direct	14-196	
Synechococcus sp. CC9902	RNase HI	NC_007513	2053018-2053761	complement	6-149	B
	RNase HII	NC_007513	1933830-1934429	complement	14-196	
Synechococcus sp. JA-2-3B'a(2-13)	RNase HI	NC_007776	1405600-1406109	direct	17-158	B
	RNase HII	NC_007776	1916462-1917103	direct	24-203	
Synechococcus sp. JA-3-3Ab	RNase HI	NC_007775	808142-808642	complement	23-164	B
	RNase HII	NC_007775	836795-837460	direct	32-211	
Synechococcus sp. WH 8102	RNase HI	NC_005070	2241126-2241872	complement	6-149	B
	RNase HII	NC_005070	2033555-2034274	complement	54-236	
Synechocystis sp. PCC 6803	RNase HI	NC_000911	2884357-2884839	direct	5-147	B
	RNase HII	NC_000911	95236-95808	direct	2-184	
Thermosynechococcus elongatus BP-1	RNase HI	NC_004113	282505-282984	direct	2-145	B
	RNase HII	NC_004113	680385-681029	complement	21-199	
**Deinococcus-Thermus**						
Deinococcus geothermalis DSM 11300	RNase HI	NC_008025	2071676-2072131	complement	5-146	B
	RNase HI	NC_008025	125304-126044	complement	2-133	
	RNase HII	NC_008025	1725635-1726309	direct	40-216	

B) List of RNase H genes in bacteria (Continued)

Species	Type	Accession No.	ORF	Direction	Domain	Combination
**Deinococcus-Thermus**						
Deinococcus radiodurans R1	RNase HI	NC_001263	907071-907610	direct	34-175	B
	RNase HI	NC_001263	443926-444657	direct	2-153	
	RNase HII	NC_001263	1971295-1971933	direct	25-201	
Thermus thermophilus HB27	RNase HI	NC_005835	1155420-1155920	direct	6-147	B
	RNase HII	NC_005835	1695850-1696461	direct	18-192	
Thermus thermophilus HB8	RNase HI	NC_006461	1480725-1481225	direct	6-147	B
	RNase HII	NC_006461	193335-193946	complement	18-192	
**Firmicutes**						
Aster yellows witches'-broom phytoplasma AYWB	RNase HIII	NC_007716	122118-123110	direct	120-325	G
Bacillus anthracis str. Ames	RNase HI	NC_003997	1540938-1541324	complement	2-128	A
	RNase HI	NC_003997	1543121-1543738	direct	57-205	
	RNase HII	NC_003997	3653759-3654532	complement	75-251	
	RNase HIII	NC_003997	4362608-4363543	direct	98-303	
Bacillus anthracis str. 'Ames Ancestor'	RNase HI	NC_007530	1541061-1541447	complement	2-128	A
	RNase HI	NC_007530	1543244-1543861	direct	57-205	
	RNase HII	NC_007530	3653886-3654659	complement	75-251	
	RNase HIII	NC_007530	4362735-4363670	direct	98-303	
Bacillus anthracis str. Sterne	RNase HI	NC_005945	1541015-1541401	complement	2-128	A
	RNase HI	NC_005945	1543156-1543815	direct	71-219	
	RNase HII	NC_005945	3654454-3655227	complement	75-251	
	RNase HIII	NC_005945	4363297-4364232	direct	98-303	
Bacillus cereus ATCC 10987	RNase HI	NC_003909	1666652-1667038	complement	2-128	A
	RNase HI	NC_003909	1668802-1669461	direct	71-219	
	RNase HII	NC_003909	3629157-3629930	complement	75-251	
	RNase HIII	NC_003909	4338186-4339121	direct	98-303	
Bacillus cereus ATCC 14579	RNase HI	NC_004722	1558450-1558836	complement	2-128	A
	RNase HI	NC_004722	1560599-1561258	direct	71-219	
	RNase HII	NC_004722	3814590-3815363	complement	75-251	
	RNase HIII	NC_004722	4502003-4502938	direct	98-303	
Bacillus cereus E33L	RNase HI	NC_006274	1565820-1566206	complement	2-128	A
	RNase HI	NC_006274	1567970-1568629	direct	71-219	
	RNase HII	NC_006274	3734314-3735087	complement	75-251	
	RNase HIII	NC_006274	4411443-4412378	direct	98-303	
Bacillus clausii KSM-K16	RNase HI'	NC_006582	1399495-1400094	direct	74-199	B'
	RNase HI	NC_006582	2129695-2130096	complement	1-133	
	RNase HII	NC_006582	2409239-2410018	complement	72-248	
Bacillus halodurans C-125	RNase HI'	NC_002570	933504-934094	direct	69-196	B'
	RNase HI	NC_002570	1842852-1843259	direct	1-135	
	RNase HI	NC_002570	2405735-2406187	direct	1-150	
	RNase HII	NC_002570	2594397-2595188	complement	77-253	
Bacillus licheniformis ATCC 14580	RNase HI	NC_006270	2283094-2283753	complement	72-219	A
	RNase HI	NC_006270	2284520-2284918	direct	1-132	
	RNase HII	NC_006270	1781813-1782580	direct	75-251	
	RNase HIII	NC_006270	2891378-2892319	direct	99-303	
Bacillus licheniformis ATCC 14580 (DSM 13)	RNase HI	NC_006322	2283952-2284611	complement	72-219	A
	RNase HI	NC_006322	2285360-2285776	direct	6-138	
	RNase HII	NC_006322	1782661-1783428	direct	75-251	
	RNase HIII	NC_006322	2891589-2892530	direct	99-303	
Bacillus subtilis subsp. subtilis str. 168	RNase HI	NC_000964	2308164-2308718	complement	30-184	A
	RNase HI	NC_000964	2309616-2310014	direct	2-132	
	RNase HII	NC_000964	1676751-1677518	direct	75-251	
	RNase HIII	NC_000964	2925099-2926040	direct	97-301	
Bacillus thuringiensis serovar konkukian str. 97-27	RNase HI	NC_005957	1567202-1567588	complement	2-128	A
	RNase HI	NC_005957	1569352-1570011	direct	71-219	
	RNase HII	NC_005957	3675432-3676205	complement	75-251	
	RNase HIII	NC_005957	4355010-4355945	direct	98-303	
Carboxydothermus hydrogenoformans Z-2901	RNase HI	NC_007503	756994-757440	direct	1-148	B
	RNase HII	NC_007503	1276066-1276761	complement	41-218	
Clostridium acetobutylicum ATCC 824	RNase HI	NC_003030	2006983-2007720	complement	4-137	B'
	RNase HI'	NC_003030	2659515-2660237	complement	103-238	
	RNase HII	NC_003030	1907294-1908058	direct	70-252	
Clostridium perfringens str. 13	RNase HI'	NC_003366	1707913-1708542	complement	70-207	B'
	RNase HII	NC_003366	1985691-1986509	complement	90-270	
Clostridium tetani E88	RNase HI'	NC_004557	2281472-2282092	complement	68-203	B'
	RNase HII	NC_004557	1343746-1344561	direct	87-269	
Desulfitobacterium hafniense Y51	RNase HI'	NC_007907	2075150-2075770	complement	71-203	B'
	RNase HII	NC_007907	2957362-2958144	complement	76-253	
Enterococcus faecalis V583	RNase HI	NC_004668	1677525-1677932	direct	2-135	A
	RNase HII	NC_004668	1606528-1607295	complement	75-251	
	RNase HIII	NC_004668	1233959-1234876	complement	94-298	
Geobacillus kaustophilus HTA426	RNase HI	NC_006510	1372704-1373369	direct	71-221	A
	RNase HII	NC_006510	1223467-1224249	direct	74-250	
	RNase HIII	NC_006510	2717612-2718547	direct	96-301	

B) List of RNase H genes in bacteria (Continued)

Species	Type	Accession No.	ORF	Direction	Domain	Combination
**Firmicutes**						
Lactobacillus acidophilus NCFM	RNase HI'	NC_006814	116459-117205	direct	91-245	B'
	RNase HII	NC_006814	952980-953732	direct	69-245	
Lactobacillus delbrueckii subsp. bulgaricus ATCC 11842	RNase HI'	NC_008054	146497-147264	direct	93-253	B'
	RNase HII	NC_008054	1080827-1081597	complement	70-246	
Lactobacillus johnsonii NCC 533	RNase HI'	NC_005362	118550-119281	direct	86-240	B'
	RNase HII	NC_005362	1011011-1011763	direct	69-245	
Lactobacillus plantarum WCFS1	RNase HI'	NC_004567	2310574-2311470	direct	70-225	B'
	RNase HI	NC_004567	1609565-1609951	complement	1-128	
	RNase HII	NC_004567	1674735-1675502	complement	73-249	
Lactobacillus sakei subsp. sakei 23K	RNase HI	NC_007576	932116-932499	complement	2-127	A
	RNase HII	NC_007576	986422-987183	complement	73-249	
	RNase HIII	NC_007576	399024-399941	complement	93-297	
Lactobacillus salivarius subsp. salivarius UCC118	RNase HI'	NC_007929	459722-460381	direct	59-216	B'
	RNase HI	NC_007929	858179-858565	complement	2-128	
	RNase HII	NC_007929	763125-763892	direct	73-249	
Lactococcus lactis subsp. lactis Il1403	RNase HII	NC_002662	1328294-1329070	complement	74-250	C
	RNase HIII	NC_002662	2349438-2350316	direct	79-283	
Listeria innocua Clip11262	RNase HI	NC_003212	2008260-2008661	direct	1-133	A
	RNase HII	NC_003212	1305326-1306111	direct	74-250	
	RNase HIII	NC_003212	1204267-1205193	complement	94-299	
Listeria monocytogenes EGD-e	RNase HI	NC_003210	1953836-1954237	direct	1-133	A
	RNase HII	NC_003210	1295772-1296557	direct	74-250	
	RNase HIII	NC_003210	1253379-1254305	complement	94-299	
Listeria monocytogenes str. 4b F2365	RNase HI	NC_002973	1934818-1935219	direct	1-133	A
	RNase HII	NC_002973	1278090-1278875	direct	74-250	
	RNase HIII	NC_002973	1230497-1231378	complement	79-284	
Mesoplasma florum L1	RNase HI'	NC_006055	559864-560484	complement	63-199	B'
	RNase HII	NC_006055	633730-634356	complement	23-200	
Moorella thermoacetica ATCC 39073	RNase HI	NC_007644	643380-643838	direct	1-142	B
	RNase HII	NC_007644	1005115-1005735	direct	30-205	
Mycoplasma capricolum subsp. capricolum ATCC 27343	RNase HI'	NC_007633	382602-383222	direct	63-198	B'
	RNase HII	NC_007633	651769-652392	complement	21-199	
Mycoplasma gallisepticum R	RNase HIII	NC_004829	555861-556883	direct	120-324	G
Mycoplasma genitalium G37	RNase HIII	NC_000908	236593-237300	complement	12-226	G
Mycoplasma hyopneumoniae 232	RNase HIII	NC_006360	766317-767063	direct	16-233	G
Mycoplasma hyopneumoniae 7448	RNase HIII	NC_007332	787986-788729	direct	15-232	G
Mycoplasma hyopneumoniae J	RNase HIII	NC_007295	768394-769137	direct	15-232	G
*Mycoplasma mobile 163*K	RNase HII	NC_006908	770837-771454	direct	25-203	C
	RNase HIII	NC_006908	179287-179970	direct	9-218	
Mycoplasma mycoides subsp. mycoides SC str. PG1	RNase HI'	NC_005364	375791-376408	direct	62-198	B'
	RNase HII	NC_005364	483996-484619	direct	21-199	
Mycoplasma penetrans HF-2	RNase HI'	NC_004432	1267458-1268120	complement	73-216	B'
	RNase HII	NC_004432	1333337-1333993	complement	31-210	
Mycoplasma pneumoniae M129	RNase HIII	NC_000912	151802-152512	complement	12-228	G
Mycoplasma pulmonis UAB CTIP	RNase HIII	NC_002771	349429-350160	complement	22-226	G
Mycoplasma synoviae 53	RNase HIII	NC_007294	734647-735360	direct	19-232	G
Oceanobacillus iheyensis HTE831	RNase HI	NC_004193	2289171-2289833	complement	70-220	A
	RNase HI	NC_004193	2511733-2512125	direct	2-130	
	RNase HII	NC_004193	1582431-1583216	direct	74-251	
	RNase HIII	NC_004193	2162847-2163773	direct	95-301	
Onion yellows phytoplasma OY-M	RNase HIII	NC_005303	722519-723511	complement	120-325	G
Staphylococcus aureus RF122	RNase HI	NC_007622	1402728-1403129	direct	4-133	A
	RNase HII	NC_007622	1197873-1198640	direct	75-251	
	RNase HIII	NC_007622	1086900-1087856	complement	104-308	
Staphylococcus aureus subsp. aureus COL	RNase HI	NC_002951	1479407-1479808	direct	4-133	A
	RNase HII	NC_002951	1269776-1270543	direct	75-251	
	RNase HIII	NC_002951	1159176-1160114	complement	98-302	
Staphylococcus aureus subsp. aureus MRSA252	RNase HI	NC_002952	1502427-1502828	direct	4-133	A
	RNase HII	NC_002952	1272222-1272989	direct	75-251	
	RNase HIII	NC_002952	1160194-1161132	complement	98-302	
Staphylococcus aureus subsp. aureus MSSA476	RNase HI	NC_002953	1469635-1470036	direct	4-133	A
	RNase HII	NC_002953	1259015-1259782	direct	75-251	
	RNase HIII	NC_002953	1148225-1149163	complement	98-302	
Staphylococcus aureus subsp. aureus Mu50	RNase HI	NC_002758	1513880-1514281	direct	4-133	A
	RNase HII	NC_002758	1306712-1307479	direct	75-251	
	RNase HIII	NC_002758	1194533-1195471	complement	98-302	
Staphylococcus aureus subsp. aureus MW2	RNase HI	NC_003923	1441080-1441481	direct	4-133	A
	RNase HII	NC_003923	1230520-1231287	direct	75-251	
	RNase HIII	NC_003923	1119439-1120377	complement	98-302	
Staphylococcus aureus subsp. aureus N315	RNase HI	NC_002745	1437468-1437869	direct	4-133	A
	RNase HII	NC_002745	1230383-1231150	direct	75-251	
	RNase HIII	NC_002745	1118205-1119143	complement	98-302	
Staphylococcus aureus subsp. aureus NCTC 8325	RNase HI	NC_007795	1375631-1376032	direct	4-133	A
	RNase HII	NC_007795	1166010-1166777	direct	75-251	
	RNase HIII	NC_007795	1055514-1056452	complement	98-302	
Staphylococcus aureus subsp. aureus USA300	RNase HI	NC_007793	1456351-1456752	direct	4-133	A
	RNase HII	NC_007793	1245956-1246723	direct	75-251	
	RNase HIII	NC_007793	1135638-1136576	complement	98-302	

B) List of RNase H genes in bacteria (Continued)

Species	Type	Accession No.	ORF	Direction	Domain	Combination
**Firmicutes**						
Staphylococcus epidermidis ATCC 12228	RNase HI	NC_004461	1134905-1135300	direct	1-131	A
	RNase HII	NC_004461	921364-922134	direct	75-251	
	RNase HIII	NC_004461	827033-827959	complement	91-295	
Staphylococcus epidermidis RP62A	RNase HI	NC_002976	1022964-1023359	direct	1-131	A
	RNase HII	NC_002976	814956-815726	direct	75-251	
	RNase HIII	NC_002976	721102-722028	complement	91-295	
Staphylococcus haemolyticus JCSC1435	RNase HI	NC_007168	1516536-1516937	complement	1-133	A
	RNase HII	NC_007168	1724803-1725570	complement	75-251	
	RNase HIII	NC_007168	1869595-1870527	direct	94-298	
Staphylococcus saprophyticus subsp. saprophyticus ATCC 15305	RNase HI	NC_007350	1352189-1352596	complement	1-135	A
	RNase HII	NC_007350	1582764-1583534	complement	75-251	
	RNase HIII	NC_007350	1721657-1722589	direct	93-297	
Streptococcus agalactiae 2603V/R	RNase HII	NC_004116	1021785-1022546	complement	73-249	C
	RNase HIII	NC_004116	1715828-1716721	direct	84-289	
Streptococcus agalactiae A909	RNase HII	NC_007432	1079971-1080732	complement	73-249	C
	RNase HIII	NC_007432	1718458-1719351	direct	84-289	
Streptococcus agalactiae NEM316	RNase HII	NC_004368	1091574-1092335	complement	73-249	C
	RNase HIII	NC_004368	1825803-1826696	direct	84-289	
Streptococcus mutans UA159	RNase HII	NC_004350	940639-941421	direct	73-252	C
	RNase HIII	NC_004350	1764918-1765829	direct	88-293	
Streptococcus pneumoniae R6	RNase HII	NC_003098	1037149-1037928	direct	73-249	C
	RNase HIII	NC_003098	364501-365373	complement	81-284	
Streptococcus pneumoniae TIGR4	RNase HII	NC_003028	1091177-1091956	direct	73-249	C
	RNase HIII	NC_003028	383074-383955	complement	81-284	
Streptococcus pyogenes M1 GAS	RNase HII	NC_002737	954926-955717	direct	74-253	C
	RNase HIII	NC_002737	1526806-1527708	direct	86-291	
Streptococcus pyogenes MGAS10270	RNase HII	NC_008022	948234-949025	direct	74-253	C
	RNase HIII	NC_008022	1575486-1576388	direct	86-291	
Streptococcus pyogenes MGAS10394	RNase HII	NC_006086	881906-882697	direct	74-253	C
	RNase HIII	NC_006086	1574811-1575713	direct	86-291	
Streptococcus pyogenes MGAS10750	RNase HII	NC_008024	979291-980082	direct	74-253	C
	RNase HIII	NC_008024	1558947-1559849	direct	86-291	
Streptococcus pyogenes MGAS2096	RNase HII	NC_008023	918080-918871	direct	74-253	C
	RNase HIII	NC_008023	1540936-1541838	direct	86-291	
Streptococcus pyogenes MGAS315	RNase HII	NC_004070	870741-871532	direct	74-253	C
	RNase HIII	NC_004070	1590447-1591349	direct	86-291	
Streptococcus pyogenes MGAS5005	RNase HII	NC_007297	873320-874111	direct	74-253	C
	RNase HIII	NC_007297	1522577-1523479	direct	86-291	
Streptococcus pyogenes MGAS6180	RNase HII	NC_007296	875822-876538	direct	49-228	C
	RNase HIII	NC_007296	1550089-1550991	direct	86-291	
Streptococcus pyogenes MGAS8232	RNase HII	NC_003485	934842-935633	direct	74-253	C
	RNase HIII	NC_003485	1581446-1582348	direct	86-291	
Streptococcus pyogenes MGAS9429	RNase HII	NC_008021	957311-958102	direct	74-253	C
	RNase HIII	NC_008021	1517048-1517950	direct	86-291	
Streptococcus pyogenes SSI-1	RNase HII	NC_004606	1001261-1002052	direct	74-253	C
	RNase HIII	NC_004606	305521-306423	complement	86-291	
Streptococcus thermophilus CNRZ1066	RNase HII	NC_006449	822542-823324	direct	78-254	C
	RNase HIII	NC_006449	1578545-1579435	direct	83-288	
Streptococcus thermophilus LMG 18311	RNase HII	NC_006448	816103-816885	direct	78-254	C
	RNase HIII	NC_006448	1574984-1575874	direct	83-288	
Thermoanaerobacter tengcongensis MB4	RNase HI	NC_003869	1344849-1345310	direct	5-146	B
	RNase HII	NC_003869	1426331-1427056	direct	60-237	
Ureaplasma parvum serovar 3 str. ATCC 700970	RNase HIII	NC_002162	456919-457869	complement	104-309	G
**Fusobacteria**						
Fusobacterium nucleatum subsp. nucleatum ATCC 25586	RNase HI'	NC_003454	1651474-1652124	complement	70-216	B'
	RNase HII	NC_003454	2019445-2020092	complement	22-205	
**Planctomycetes**						
Rhodopirellula baltica SH 1	RNase HI	NC_005027	5552071-5552547	complement	10-155	E
**Alphaproteobacteria**						
Agrobacterium tumefaciens str. C58 Cereon	RNase HI	NC_003062	771637-772077	direct	1-141	B
	RNase HI	NC_003063	1886926-1887399	direct	2-153	
	RNase HII	NC_003062	719543-720232	complement	49-226	
Agrobacterium tumefaciens str. C58 UWash	RNase HI	NC_003304	771837-772277	direct	1-141	B
	RNase HI	NC_003305	187357-187830	complement	2-153	
	RNase HII	NC_003304	719742-720395	complement	37-214	
Anaplasma marginale str. St. Maries	RNase HI	NC_004842	105394-105909	direct	23-164	B
	RNase HII	NC_004842	224173-225192	complement	122-301	
Anaplasma phagocytophilum HZ	RNase HI	NC_007797	114020-114472	direct	3-144	B
	RNase HII	NC_007797	1081522-1082154	direct	19-198	
Bartonella henselae str. Houston-1	RNase HI	NC_005956	509648-510115	direct	5-145	B
	RNase HII	NC_005956	487066-487737	direct	35-214	
Bartonella quintana str. Toulouse	RNase HI	NC_005955	446165-446632	direct	5-145	B
	RNase HII	NC_005955	422818-423489	direct	35-214	

B) List of RNase H genes in bacteria (Continued)

Species	Type	Accession No.	ORF	Direction	Domain	Combination
**Alphaproteobacteria**						
Bradyrhizobium japonicum USDA 110	RNase HI	NC_004463	1429127-1429591	direct	4-144	B
	RNase HII	NC_004463	2754052-2754837	direct	45-221	
Brucella abortus biovar 1 str. 9-941	RNase HI	NC_006932	496760-497224	direct	1-141	B
	RNase HII	NC_006932	416786-417448	complement	35-214	
Brucella melitensis 16M	RNase HI	NC_003317	1507514-1507978	complement	1-141	B
	RNase HII	NC_003317	1586759-1587421	direct	35-214	
Brucella melitensis biovar Abortus 2308	RNase HI	NC_007618	493063-493527	direct	1-141	B
	RNase HII	NC_007618	413153-413815	complement	35-214	
Brucella suis 1330	RNase HI	NC_004310	474948-475412	direct	1-141	B
	RNase HII	NC_004310	394928-395590	complement	35-214	
Candidatus Pelagibacter ubique HTCC1062	RNase HI	NC_007205	135675-136100	direct	2-139	B
	RNase HII	NC_007205	119534-120094	direct	5-182	
Caulobacter crescentus CB15	RNase HI	NC_002696	3614808-3615257	direct	2-142	B
	RNase HII	NC_002696	399852-400469	complement	13-190	
Ehrlichia canis str. Jake	RNase HI	NC_007354	1067042-1067482	complement	5-145	B
	RNase HII	NC_007354	260433-261062	complement	22-200	
Ehrlichia chaffeensis str. Arkansas	RNase HI	NC_007799	246522-246962	direct	5-145	B
	RNase HII	NC_007799	968499-969128	direct	22-200	
Ehrlichia ruminantium str. Gardel	RNase HI	NC_006831	1204976-1205437	complement	12-152	B
	RNase HII	NC_006831	276941-277603	complement	30-210	
Ehrlichia ruminantium str. Welgevonden 1	RNase HI	NC_005295	1239485-1239925	complement	5-145	B
	RNase HII	NC_005295	300081-300719	complement	22-202	
Ehrlichia ruminantium str. Welgevonden 2	RNase HI	NC_006832	1217579-1218040	complement	12-152	B
	RNase HII	NC_006832	280074-280736	complement	30-210	
Erythrobacter litoralis HTCC2594	RNase HI	NC_007722	354264-354698	complement	1-141	B
	RNase HII	NC_007722	493323-493961	direct	21-205	
Gluconobacter oxydans 621H	RNase HI	NC_006677	193146-193598	complement	8-148	B
	RNase HII	NC_006677	366896-367519	complement	15-196	
Jannaschia sp. CCS1	RNase HI	NC_007802	499515-499988	complement	1-146	B
	RNase HII	NC_007802	394195-394794	direct	16-193	
Magnetospirillum magneticum AMB-1	RNase HI	NC_007626	819075-819539	complement	8-148	B
	RNase HII	NC_007626	4399451-4400056	complement	15-195	
Mesorhizobium loti MAFF303099	RNase HI	NC_002678	6194516-6195049	direct	2-142	B
	RNase HII	NC_002678	6129144-6129839	complement	35-214	
Neorickettsia sennetsu str. Miyayama	RNase HI	NC_007798	584101-584556	complement	1-143	B
	RNase HII	NC_007798	621093-621680	direct	12-189	
Nitrobacter hamburgensis X14	RNase HI	NC_007964	4122987-4123442	complement	6-146	B
	RNase HII	NC_007964	3550057-3550860	complement	60-236	
Nitrobacter winogradskyi Nb-255	RNase HI	NC_007406	2920007-2920471	complement	6-146	B
	RNase HII	NC_007406	2823769-2824545	complement	51-227	
Novosphingobium aromaticivorans DSM 12444	RNase HI	NC_007794	1128544-1128975	complement	1-141	B
	RNase HI	NC_007794	1553048-1553437	complement	5-129	
	RNase HII	NC_007794	1938693-1939283	direct	4-190	
Rhizobium etli CFN 42	RNase HI	NC_007761	1011173-1011628	direct	1-141	B
	RNase HI	NC_007761	2470715-2471212	complement	9-160	
	RNase HII	NC_007761	909201-909890	complement	37-214	
Rhodobacter sphaeroides 2.4.1	RNase HI	NC_007493	2617237-2617764	direct	26-170	B
	RNase HII	NC_007493	55434-56072	complement	23-200	
Rhodopseudomonas palustris BisB18	RNase HI	NC_007925	4529874-4530335	complement	4-144	B
	RNase HII	NC_007925	4863966-4864766	complement	61-237	
Rhodopseudomonas palustris BisB5	RNase HI	NC_007958	4476977-4477429	complement	1-141	B
	RNase HII	NC_007958	1365648-1366544	direct	88-264	
Rhodopseudomonas palustris CGA009	RNase HI	NC_005296	4817500-4817967	complement	6-146	B
	RNase HII	NC_005296	1135479-1136393	direct	99-275	
Rhodopseudomonas palustris HaA2	RNase HI	NC_007778	1528662-1529147	direct	12-152	B
	RNase HII	NC_007778	1240599-1241453	direct	74-250	
Rhodospirillum rubrum ATCC 11170	RNase HI	NC_007643	3517282-3517782	direct	11-151	B
	RNase HII	NC_007643	3699820-3700476	direct	15-197	
Rickettsia bellii RML369-C	RNase HI	NC_007940	412221-412688	direct	5-145	B
	RNase HII	NC_007940	572887-573477	complement	18-193	
Rickettsia conorii str. Malish 7	RNase HI	NC_003103	1029683-1030150	complement	5-145	B
	RNase HII	NC_003103	268353-268934	direct	18-193	
Rickettsia felis URRWXCal2	RNase HI	NC_007109	200109-200567	direct	2-142	B
	RNase HII	NC_007109	1122962-1123543	complement	18-193	
Rickettsia prowazekii str. Madrid E	RNase HI	NC_000963	913329-913787	complement	2-142	B
	RNase HII	NC_000963	244586-245167	direct	18-193	
Rickettsia typhi str. Wilmington	RNase HI	NC_006142	909165-909623	complement	2-142	B
	RNase HII	NC_006142	243899-244480	direct	18-193	
Silicibacter pomeroyi DSS-3	RNase HI	NC_003911	3421234-3421701	direct	1-146	B
	RNase HII	NC_003911	3656206-3656910	complement	50-227	
Silicibacter sp. TM1040	RNase HI	NC_008044	2713912-2714385	direct	1-146	B
	RNase HII	NC_008044	2785589-2786278	complement	45-222	
Sinorhizobium meliloti 1021	RNase HI	NC_003047	660004-660513	complement	12-160	B
	RNase HI	NC_003047	1000917-1001378	direct	1-141	
	RNase HII	NC_003047	919580-920230	complement	36-213	
Sphingopyxis alaskensis RB2256	RNase HI	NC_008048	1172105-1172557	complement	4-144	B
	RNase HII	NC_008048	989301-989897	direct	17-192	

B) List of RNase H genes in bacteria (Continued)

Species	Type	Accession No.	ORF	Direction	Domain	Combination
**Alphaproteobacteria**						
Wolbachia endosymbiont of *Drosophila melanogaster*	RNase HI	NC_002978	283091-283528	direct	3-143	B
	RNase HII	NC_002978	1058063-1058659	direct	17-194	
Wolbachia endosymbiont strain TRS of Brugia malayi	RNase HI	NC_006833	404094-404504	complement	2-134	B
	RNase HII	NC_006833	836656-837267	complement	17-194	
Zymomonas mobilis subsp. mobilis ZM4	RNase HI	NC_006526	1642109-1642579	direct	8-148	B
**Betaproteobacteria**						
Azoarcus sp. EbN1	RNase HI	NC_006513	3830951-3831412	complement	2-142	B
	RNase HII	NC_006513	3564193-3564807	direct	11-188	
Bordetella bronchiseptica RB50	RNase HI	NC_002927	4557048-4557515	complement	10-150	B
	RNase HII	NC_002927	2782168-2782773	direct	18-195	
Bordetella parapertussis 12822	RNase HI	NC_002928	4152822-4153289	complement	10-150	B
	RNase HII	NC_002928	1648897-1649502	direct	18-195	
Bordetella pertussis Tohama I	RNase HI	NC_002929	3423301-3423768	complement	10-150	B
	RNase HII	NC_002929	1508454-1509059	direct	18-195	
Burkholderia cenocepacia AU 1054	RNase HI	NC_008060	868593-869036	complement	4-143	B
	RNase HII	NC_008062	669557-670201	direct	29-206	
Burkholderia mallei ATCC 23344	RNase HI	NC_006348	793756-794202	complement	4-143	B
	RNase HII	NC_006348	1598457-1599101	complement	29-206	
Burkholderia pseudomallei 1710b	RNase HI	NC_007434	1680477-1680917	complement	1-141	B
	RNase HII	NC_007434	2845097-2845741	complement	29-206	
Burkholderia pseudomallei K96243	RNase HI	NC_006350	1567199-1567645	complement	4-143	B
	RNase HII	NC_006350	2575277-2575921	complement	29-206	
Burkholderia sp. 383	RNase HI	NC_007510	1384168-1384611	complement	4-143	B
	RNase HII	NC_007510	2367280-2367924	complement	29-206	
Burkholderia thailandensis E264	RNase HI	NC_007651	3206001-3206447	direct	4-143	B
	RNase HII	NC_007651	2312993-2313637	direct	29-206	
Burkholderia xenovorans LB400	RNase HI	NC_007951	3539487-3539933	direct	3-143	B
	RNase HII	NC_007951	3031865-3032611	complement	32-210	
Chromobacterium violaceum ATCC 12472	RNase HI	NC_005085	1321674-1322117	direct	4-145	B
	RNase HII	NC_005085	2380982-2381578	direct	8-186	
Dechloromonas aromatica RCB	RNase HI	NC_007298	1515702-1516352	direct	1-153	B
	RNase HI	NC_007298	1709113-1709559	complement	4-144	
	RNase HI	NC_007298	3953143-3953634	direct	29-163	
	RNase HII	NC_007298	1887042-1887632	direct	12-189	
Methylobacillus flagellatus KT	RNase HI	NC_007947	1586361-1586816	complement	3-143	B
	RNase HII	NC_007947	1620989-1621570	complement	9-186	
Neisseria gonorrhoeae FA 1090	RNase HI	NC_002946	1102249-1102686	direct	2-142	B
	RNase HII	NC_002946	1761924-1762508	direct	6-184	
Neisseria meningitidis MC58	RNase HI	NC_003112	1681632-1682069	direct	2-142	B
	RNase HII	NC_003112	190697-191281	complement	6-184	
Neisseria meningitidis Z2491	RNase HI	NC_003116	1764323-1764760	direct	2-142	B
	RNase HII	NC_003116	73825-74409	direct	6-184	
Nitrosomonas europaea ATCC 19718	RNase HI	NC_004757	165523-166008	direct	7-153	B
	RNase HII	NC_004757	1842393-1843046	complement	20-198	
Nitrosospira multiformis ATCC 25196	RNase HI	NC_007614	1859121-1859588	complement	6-146	B
	RNase HII	NC_007614	764134-764733	direct	10-187	
Polaromonas sp. JS666	RNase HI	NC_007948	5118574-5119011	direct	1-145	B
	RNase HI	NC_007948	2371565-2372044	direct	11-153	
	RNase HII	NC_007948	2822407-2823006	complement	17-194	
Ralstonia eutropha JMP134	RNase HI	NC_007347	2417285-2417722	direct	1-141	B
	RNase HII	NC_007347	2043300-2043956	complement	26-207	
Ralstonia metallidurans CH34	RNase HI	NC_007973	2420845-2421282	direct	1-141	B
	RNase HII	NC_007973	1570802-1571482	direct	27-207	
Ralstonia solanacearum GMI1000	RNase HI	NC_003295	1614866-1615321	complement	1-141	B
	RNase HII	NC_003295	1518122-1518832	direct	30-210	
Rhodoferax ferrireducens T118	RNase HI	NC_007908	1574165-1574614	direct	1-143	B
	RNase HII	NC_007908	2158121-2158744	direct	21-198	
Thiobacillus denitrificans ATCC 25259	RNase HI	NC_007404	1747503-1747949	complement	3-143	B
	RNase HII	NC_007404	841944-842558	direct	17-194	
**Deltaproteobacteria**						
Anaeromyxobacter dehalogenans 2CP-C	RNase HI	NC_007760	838264-838770	direct	33-168	B
	RNase HI	NC_007760	2339153-2339704	complement	2-151	
	RNase HII	NC_007760	324321-325172	direct	81-258	
Bdellovibrio bacteriovorus HD100	RNase HI	NC_005363	2044076-2044537	complement	14-151	B'
	RNase HI'	NC_005363	3036447-3037238	complement	18-164	
	RNase HII	NC_005363	2025670-2026335	complement	39-217	
Desulfotalea psychrophila LSv54	RNase HI'	NC_006138	1023218-1024003	direct	105-247	B'
	RNase HII	NC_006138	3163253-3163930	direct	31-211	
Desulfovibrio desulfuricans G20	RNase HI	NC_007519	2920672-2921136	complement	1-142	B
	RNase HII	NC_007519	1123753-1124544	complement	42-248	
Desulfovibrio vulgaris subsp. vulgaris str. Hildenborough	RNase HI	NC_002937	763517-763987	direct	3-144	B
	RNase HII	NC_002937	923544-924206	complement	19-207	
Geobacter metallireducens GS-15	RNase HI	NC_007517	1038344-1038802	direct	2-142	B
	RNase HII	NC_007517	3249333-3249974	complement	30-207	

B) List of RNase H genes in bacteria (Continued)

Species	Type	Accession No.	ORF	Direction	Domain	Combination
**Deltaproteobacteria**						
Geobacter sulfurreducens PCA	RNase HI	NC_002939	2272223-2272672	complement	2-142	B
	RNase HII	NC_002939	680763-681416	direct	30-207	
Lawsonia intracellularis PHE/MN1-00	RNase HI	NC_008011	943310-943795	complement	6-147	B
	RNase HII	NC_008011	279395-280069	direct	34-221	
Myxococcus xanthus DK 1622	RNase HI	NC_008095	7095320-7095763	direct	14-147	B'
	RNase HI'	NC_008095	2628106-2628873	complement	3-154	
	RNase HII	NC_008095	2994879-2995760	complement	84-262	
Pelobacter carbinolicus DSM 2380	RNase HI	NC_007498	262583-263041	direct	5-145	B
	RNase HI	NC_007498	2654453-2654956	complement	4-152	
	RNase HI	NC_007498	3349400-3350842	direct	289-427	
	RNase HII	NC_007498	2592294-2592923	complement	28-205	
Syntrophus aciditrophicus SB	RNase HI	NC_007759	2740502-2741023	direct	21-162	B
	RNase HII	NC_007759	2374501-2375139	complement	20-197	
**Epsilonproteobacteria**						
Campylobacter jejuni RM1221	RNase HI	NC_003912	1702771-1703211	complement	1-136	B
	RNase HII	NC_003912	14088-14663	complement	19-191	
Campylobacter jejuni subsp. jejuni NCTC 11168	RNase HI	NC_002163	1561472-1561912	complement	1-136	B
	RNase HII	NC_002163	15844-16419	complement	19-191	
Helicobacter hepaticus ATCC 51449	RNase HI	NC_004917	678422-678868	direct	3-138	B
	RNase HII	NC_004917	6282-6905	complement	4-206	
Helicobacter pylori 26695	RNase HI	NC_000915	709533-709964	direct	1-136	B
	RNase HII	NC_000915	1383299-1383928	complement	8-193	
Helicobacter pylori HPAG1	RNase HI	NC_008086	674125-674556	direct	1-136	B
	RNase HII	NC_008086	1315480-1316094	complement	3-188	
Helicobacter pylori J99	RNase HI	NC_000921	671961-672392	direct	1-136	B
	RNase HII	NC_000921	1361044-1361673	complement	8-193	
Thiomicrospira denitrificans ATCC 33889	RNase HI	NC_007575	1174933-1175451	direct	8-156	B
	RNase HI	NC_007575	2106542-2106973	complement	1-136	
	RNase HII	NC_007575	298260-298826	complement	6-177	
Wolinella succinogenes DSM 1740	RNase HI	NC_005090	1792079-1792498	complement	1-136	B
	RNase HII	NC_005090	1618320-1618871	direct	4-175	
**Gammaproteobacteria**						
Acinetobacter sp. ADP1	RNase HI	NC_005966	1124144-1125496	complement	2-137	B
	RNase HII	NC_005966	1247459-1248028	direct	3-181	
Baumannia cicadellinicola str. Hc (Homalodisca coagulata)	RNase HI	NC_007984	488425-488886	direct	2-142	E
Buchnera aphidicola str. Bp (Baizongia pistaciae)	RNase HI	NC_004545	269553-270014	complement	2-142	E
Buchnera aphidicola str. Sg (Schizaphis graminum)	RNase HI	NC_004061	278071-278556	complement	2-142	E
Candidatus Blochmannia floridanus	RNase HI	NC_005061	239641-240144	complement	2-143	B
	RNase HII	NC_005061	310493-311110	direct	16-198	
Candidatus Blochmannia pennsylvanicus str. BPEN	RNase HI	NC_007292	261986-262441	complement	2-143	B
	RNase HII	NC_007292	339451-340104	direct	28-206	
Chromohalobacter salexigens DSM 3043	RNase HI	NC_007963	2197806-2198300	complement	10-149	B
	RNase HII	NC_007963	639686-640288	direct	17-194	
Colwellia psychrerythraea 34H	RNase HI'	NC_003910	1743847-1744665	complement	98-247	B'
	RNase HII	NC_003910	1612598-1613251	direct	19-208	
Coxiella burnetii RSA 493	RNase HI	NC_002971	282596-283060	direct	6-146	B
	RNase HII	NC_002971	1287713-1288321	complement	15-192	
Erwinia carotovora subsp. atroseptica SCRI1043	RNase HI	NC_004547	3749751-3750215	direct	2-142	B
	RNase HII	NC_004547	1168997-1169593	direct	14-191	
Escherichia coli CFT073	RNase HI	NC_004431	247158-247736	complement	39-179	B
	RNase HII	NC_004431	214528-215124	direct	13-190	
Escherichia coli K12	RNase HI	NC_000913	235535-236002	complement	2-142	B
	RNase HII	NC_000913	204493-205089	direct	13-190	
Escherichia coli O157:H7 EDL933	RNase HI	NC_002655	239086-239553	complement	2-142	B
	RNase HII	NC_002655	207837-208433	direct	13-190	
Escherichia coli O157:H7 str. Sakai	RNase HI	NC_002695	239085-239552	complement	2-142	B
	RNase HII	NC_002695	207836-208432	direct	13-190	
Escherichia coli UTI89	RNase HI	NC_007946	240503-241081	complement	39-179	B
	RNase HII	NC_007946	209294-209890	direct	13-190	
Escherichia coli W3110	RNase HI	AC_000091	235535-236002	complement	2-142	B
	RNase HII	AC_000091	204493-205089	direct	13-190	
Francisella tularensis subsp. holarctica	RNase HI	NC_007880	835373-835831	direct	7-147	B
	RNase HII	NC_007880	1132053-1132616	direct	4-181	
Francisella tularensis subsp. tularensis SCHU S4	RNase HI	NC_006570	609632-610114	direct	15-155	B
	RNase HII	NC_006570	1299107-1299670	complement	4-181	
Haemophilus ducreyi 35000HP	RNase HI	NC_002940	981501-981962	direct	1-141	B
	RNase HII	NC_002940	816071-816664	complement	14-191	
Haemophilus influenzae 86-028NP	RNase HI	NC_007146	214457-214921	direct	2-142	B
	RNase HII	NC_007146	1161534-1162127	complement	12-190	
Haemophilus influenzae Rd KW20	RNase HI	NC_000907	153323-153787	direct	2-142	B
	RNase HII	NC_000907	1124264-1124857	complement	12-190	
Hahella chejuensis KCTC 2396	RNase HI	NC_007645	2591814-2592260	direct	1-141	B
	RNase HII	NC_007645	5381677-5382291	complement	20-197	
Idiomarina loihiensis L2TR	RNase HI	NC_006512	1822411-1822884	direct	5-144	B
	RNase HII	NC_006512	1790050-1790613	complement	3-180	

B) List of RNase H genes in bacteria (Continued)

Species	Type	Accession No.	ORF	Direction	Domain	Combination
**Gammaproteobacteria**						
Legionella pneumophila str. Lens	RNase HI	NC_006369	1492925-1493356	direct	1-140	B
	RNase HII	NC_006369	1481100-1481675	direct	10-185	
Legionella pneumophila str. Paris	RNase HI	NC_006368	1494513-1494944	direct	1-140	B
	RNase HII	NC_006368	1481387-1481962	direct	10-185	
Legionella pneumophila subsp. pneumophila str. Philadelphia 1	RNase HI	NC_002942	1531442-1531873	direct	1-140	B
	RNase HII	NC_002942	1519616-1520191	direct	10-185	
Mannheimia succiniciproducens MBEL55E	RNase HI	NC_006300	1566286-1566762	direct	6-146	B
	RNase HII	NC_006300	389788-390384	direct	14-191	
Methylococcus capsulatus str. Bath	RNase HI	NC_002977	759414-759881	direct	5-145	B
	RNase HII	NC_002977	2624340-2624921	complement	6-183	
Nitrosococcus oceani ATCC 19707	RNase HI	NC_007484	3191535-3191978	complement	2-142	B
	RNase HII	NC_007484	2334508-2335113	complement	18-195	
Pasteurella multocida subsp. multocida str. Pm70	RNase HI	NC_002663	132840-133304	direct	2-142	B
	RNase HII	NC_002663	2239228-2239821	direct	13-190	
Photobacterium profundum SS9	RNase HI'	NC_006370	2161121-2161870	complement	77-225	B'
	RNase HI	NC_006370	3350860-3351399	direct	25-166	
	RNase HII	NC_006370	3387343-3387948	complement	17-194	
Photorhabdus luminescens subsp. laumondii TTO1	RNase HI	NC_005126	1084187-1084657	complement	2-142	B
	RNase HII	NC_005126	786990-787580	direct	12-189	
Pseudoalteromonas atlantica T6c	RNase HI	NC_008228	2880192-2880653	direct	1-141	B
	RNase HI	NC_008228	4074405-4074896	complement	1-159	
	RNase HII	NC_008228	1512654-1513247	direct	6-183	
Pseudoalteromonas haloplanktis TAC125	RNase HI	NC_007481	2068513-2068977	direct	2-142	B
	RNase HII	NC_007481	2129841-2130443	complement	12-189	
Pseudomonas aeruginosa PAO1	RNase HI	NC_002516	1972959-1973405	direct	4-144	B
	RNase HII	NC_002516	4080439-4081044	complement	15-192	
Pseudomonas entomophila L48	RNase HI	NC_008027	3840496-3840942	complement	2-142	B
	RNase HII	NC_008027	4488928-4489551	complement	15-192	
Pseudomonas fluorescens Pf-5	RNase HI	NC_004129	3804857-3805309	complement	2-142	B
	RNase HII	NC_004129	1361904-1362557	direct	25-202	
Pseudomonas fluorescens PfO-1	RNase HI	NC_007492	2470094-2470555	direct	5-145	B
	RNase HII	NC_007492	1289051-1289692	direct	23-200	
Pseudomonas putida KT2440	RNase HI	NC_002947	4679508-4679954	complement	2-142	B
	RNase HII	NC_002947	1799203-1799826	direct	15-192	
Pseudomonas syringae pv. phaseolicola 1448A	RNase HI	NC_005773	1981160-1981609	direct	2-142	B
	RNase HII	NC_005773	4380953-4381621	complement	20-197	
Pseudomonas syringae pv. syringae B728a	RNase HI	NC_007005	2004697-2005149	direct	2-142	B
	RNase HII	NC_007005	1537295-1537951	direct	16-193	
Pseudomonas syringae pv. tomato str. DC3000	RNase HI	NC_004578	4189457-4189909	complement	2-142	B
	RNase HII	NC_004578	1707663-1708316	direct	15-192	
Psychrobacter arcticus 273-4	RNase HI	NC_007204	1000960-1002543	complement	24-165	B
	RNase HII	NC_007204	1094240-1095103	direct	64-281	
Psychrobacter cryohalolentis K5	RNase HI	NC_007969	1011925-1013523	complement	24-165	B
	RNase HII	NC_007969	1849337-1850197	complement	64-280	
Saccharophagus degradans 2-40	RNase HI'	NC_007912	82187-82945	complement	81-228	B'
	RNase HI	NC_007912	4381311-4381808	complement	1-153	
	RNase HII	NC_007912	3277856-3278455	complement	12-189	
Salmonella enterica subsp. enterica serovar Choleraesuis str. SC-B67	RNase HI	NC_006905	298780-299604	complement	121-261	B
	RNase HII	NC_006905	264922-265518	direct	13-190	
Salmonella enterica subsp. enterica serovar Paratyphi A str. ATCC 9150	RNase HI	NC_006511	2605688-2606155	direct	2-142	B
	RNase HII	NC_006511	273074-273670	direct	13-190	
Salmonella enterica subsp. enterica serovar Typhi str. CT18	RNase HI	NC_003198	300696-301163	complement	2-142	B
	RNase HII	NC_003198	265810-266406	direct	13-190	
Salmonella enterica subsp. enterica serovar Typhi Ty2	RNase HI	NC_004631	2678918-2679385	direct	2-142	B
	RNase HII	NC_004631	265801-266397	direct	13-190	
Salmonella typhimurium LT2	RNase HI	NC_003197	302871-303338	complement	2-142	B
	RNase HII	NC_003197	268928-269524	direct	13-190	
Shewanella denitrificans OS217	RNase HI'	NC_007954	880428-881219	direct	91-239	B'
	RNase HI	NC_007954	2395224-2395703	direct	5-145	
	RNase HII	NC_007954	1822231-1822875	direct	27-208	
Shewanella oneidensis MR-1	RNase HI	NC_004347	2690175-2690651	direct	4-144	B
	RNase HII	NC_004347	1725561-1726190	direct	21-203	
Shigella boydii Sb227	RNase HI	NC_007613	221490-221957	complement	2-142	B
	RNase HII	NC_007613	190510-191106	direct	13-190	
Shigella dysenteriae Sd197	RNase HI	NC_007606	236700-237167	complement	2-142	B
	RNase HII	NC_007606	204755-205351	direct	13-190	
Shigella flexneri 2a str. 2457T	RNase HI	NC_004741	227566-228033	complement	2-142	B
	RNase HII	NC_004741	194924-195520	direct	13-190	
Shigella flexneri 2a str. 301	RNase HI	NC_004337	228437-229015	complement	39-179	B
	RNase HII	NC_004337	195437-196033	direct	13-190	
Shigella sonnei Ss046	RNase HI	NC_007384	251973-252440	complement	2-142	B
	RNase HII	NC_007384	219414-220010	direct	13-190	
Sodalis glossinidius str. 'morsitans'	RNase HI	NC_007712	998648-999139	complement	2-142	B
	RNase HII	NC_007712	3333310-3333906	complement	14-191	
Thiomicrospira crunogena XCL-2	RNase HI	NC_007520	1005816-1006253	direct	1-141	B
	RNase HII	NC_007520	1387931-1388554	complement	21-197	
Vibrio cholerae O1 biovar eltor str. N16961	RNase HI	NC_002505	530684-531124	direct	1-144	B
	RNase HI	NC_002505	2388824-2389294	direct	2-142	
	RNase HII	NC_002505	2401250-2401870	complement	22-199	

B) List of RNase H genes in bacteria (Continued)

Species	Type	Accession No.	ORF	Direction	Domain	Combination
**Gammaproteobacteria**						
Vibrio fischeri ES114	RNase HI	NC 006840	2171052-2171525	direct	6-146	B
	RNase HI	NC_006841	598303-598749	direct	4-147	
	RNase HII	NC_006840	2183513-2184136	complement	23-200	
Vibrio parahaemolyticus RIMD 2210633	RNase HI	NC 004603	672008-672505	direct	15-162	B
	RNase HI	NC_004603	2403675-2404139	direct	2-142	
	RNase HI	NC_004605	429180-429689	direct	15-162	
	RNase HII	NC_004603	2416025-2416660	complement	27-204	
Vibrio vulnificus CMCP6	RNase HI	NC 004459	1880594-1881061	complement	2-142	B
	RNase HII	NC_004459	1868328-1868951	direct	22-199	
Vibrio vulnificus YJ016	RNase HI	NC 005139	503675-504112	direct	1-144	B
	RNase HI	NC_005139	2560072-2560539	direct	2-142	
	RNase HII	NC_005139	2572181-2572804	complement	22-199	
Wigglesworthia glossinidia endosymbiont of Glossina brevipalpis	RNase HI	NC 004344	74186-74659	complement	2-142	E
Xanthomonas axonopodis pv. citri str. 306	RNase HI	NC 003919	1243888-1244340	direct	1-141	B
	RNase HII	NC_003919	1621794-1622528	complement	34-213	
Xanthomonas campestris pv. campestris str. 8004	RNase HI	NC 007086	3898212-3898664	complement	1-141	B
	RNase HII	NC_007086	3459761-3460483	direct	34-213	
Xanthomonas campestris pv. campestris str. ATCC 33913	RNase HI	NC 003902	1141885-1142337	direct	1-141	B
	RNase HII	NC_003902	1583298-1584020	complement	34-213	
Xanthomonas campestris pv. vesicatoria str. 85-10	RNase HI	NC 007508	1224800-1225252	direct	1-141	E
Xanthomonas oryzae pv. oryzae KACC10331	RNase HI	NC 006834	1060760-1061212	direct	1-141	B
	RNase HII	NC_006834	2063703-2064461	complement	44-223	
Xanthomonas oryzae pv. oryzae MAFF 311018	RNase HI	NC 007705	1028062-1028514	direct	1-141	B
	RNase HII	NC_007705	2043807-2044547	complement	38-217	
Xylella fastidiosa 9a5c	RNase HI	NC 002488	2048713-2049174	complement	4-144	B
	RNase HII	NC_002488	1000079-1000783	complement	19-198	
Xylella fastidiosa Temecula1	RNase HI	NC 004556	1418930-1419382	complement	1-141	B
	RNase HII	NC_004556	413390-414094	complement	19-198	
Yersinia pestis Antiqua	RNase HI	NC 008150	645433-645897	complement	2-142	B
	RNase HII	NC_008150	616856-617452	direct	14-191	
Yersinia pestis biovar Medievalis str. 91001	RNase HI	NC 005810	3073932-3074396	direct	2-142	B
	RNase HII	NC_005810	3102382-3102978	complement	14-191	
Yersinia pestis CO92	RNase HI	NC 003143	1228698-1229162	complement	2-142	B
	RNase HII	NC_003143	1200120-1200716	direct	14-191	
Yersinia pestis KIM	RNase HI	NC 004088	3405646-3406110	direct	2-142	B
	RNase HII	NC_004088	3434092-3434688	complement	14-191	
Yersinia pestis Nepal516	RNase HI	NC 008149	3274713-3275177	direct	2-142	B
	RNase HII	NC_008149	3303159-3303755	complement	14-191	
Yersinia pseudotuberculosis IP 32953	RNase HI	NC 006155	3498912-3499376	direct	2-142	B
	RNase HII	NC_006155	3527439-3528035	complement	14-191	
**Spirochaetes**						
Borrelia burgdorferi B31	RNase HI'	NC 001318	897096-897740	complement	74-212	B'
	RNase HII	NC_001318	45446-45991	direct	3-181	
Borrelia garinii PBi	RNase HI'	NC 006156	899069-899668	complement	58-196	B'
	RNase HII	NC_006156	45306-45851	direct	3-181	
Leptospira interrogans serovar Copenhageni str. Fiocruz L1-130	RNase HI	NC 005823	2334943-2335332	direct	1-129	B
	RNase HII	NC_005823	1921387-1922082	direct	24-221	
Leptospira interrogans serovar Lai str. 56601	RNase HI	NC 004342	1947579-1947968	complement	2-129	B
	RNase HII	NC_004342	2359353-2360048	complement	24-221	
Treponema denticola ATCC 35405	RNase HI	NC 002967	2597515-2597997	direct	1-157	B
	RNase HII	NC_002967	1591264-1591860	direct	3-196	
Treponema pallidum subsp. pallidum str. Nichols	RNase HI	NC 000919	377198-377707	complement	4-159	E
**Thermotogae**						
Thermotoga maritima MSB8	RNase HI'	NC 000853	1322788-1323459	complement	63-197	B'
	RNase HII	NC_000853	933029-933745	complement	15-188	
