# Supplementary material for: Evolution of ribonuclease H genes in prokaryotes to avoid inheritance of redundant genes
Source: BMC Evol Biol. 2007 Jul 31;7:128. doi: 10.1186/1471-2148-7-128 (PMC1950709; doi:10.1186/1471-2148-7-128)
Supplement: Additional file 2 — RNase HI sequences of the bacillales and lactobacillales used for the multiple alignments. [file 1471-2148-7-128-S2.pdf]

Additional file 2: RNase HI sequences of bacillales and lactobacillales used for the multiple alignments. ORF numbers indicate the genomic positions of the genes that encode RNase HI. Domain numbers indicate the amino acid positions relative to the start of each protein sequence. The RNase H combination refers to the groups defined in Figure 1. Apostrophes (i.e., B') represent the presence of dsRHbd.

Species	Type	Accession No.	ORF	Direction	Domain	Combination
Escherichia coli K12	RNase HI	NC_000913	235535-236002	complement	2-142	B
Lactobacillus delbrueckii subsp. bulgaricus ATCC 11842	RNase HI'	NC_008054	146497-147264	direct	93-253	B'
Lactobacillus salivarius subsp. salivarius UCC118	RNase HI'	NC_007929	459722-460381	direct	59-216	B'
Lactobacillus plantarum WCFS1	RNase HI'	NC_004567	2310574-2311470	direct	70-225	B'
Lactobacillus acidophilus NCFM	RNase HI'	NC_006814	116459-117205	direct	91-245	B'
Lactobacillus johnsonii NCC 533	RNase HI'	NC_005362	118550-119281	direct	86-240	B'
Lactobacillus sakei subsp. sakei 23K	RNase HI	NC_007576	932116-932499	complement	2-127	A
Enterococcus faecalis V583	RNase HI	NC_004668	1677525-1677932	direct	2-135	A
Staphylococcus saprophyticus subsp. saprophyticus ATCC 15305	RNase HI	NC_007350	1352189-1352596	complement	1-135	A
Staphylococcus aureus subsp. aureus N315	RNase HI	NC_002745	1437468-1437869	direct	4-133	A
Staphylococcus haemolyticus JCSC1435	RNase HI	NC_007168	1516536-1516937	complement	1-133	A
Staphylococcus epidermidis RP62A	RNase HI	NC_002976	1022964-1023359	direct	1-131	A
Oceanobacillus iheyensis HTE831	RNase HI	NC_004193	2511733-2512125	direct	2-130	A
Bacillus cereus ATCC 10987	RNase HI	NC_003909	1666652-1667038	complement	2-128	A
Bacillus thuringiensis serovar konkukian str. 97-27	RNase HI	NC_005957	1567202-1567588	complement	2-128	A
Bacillus anthracis str. Ames	RNase HI	NC_003997	1540938-1541324	complement	2-128	A
Bacillus licheniformis ATCC 14580	RNase HI	NC_006270	2284520-2284918	direct	1-132	A
Bacillus subtilis subsp. subtilis str. 168	RNase HI	NC_000964	2309616-2310014	direct	2-132	A
Listeria innocua Clip11262	RNase HI	NC_003212	2008260-2008661	direct	1-133	A
Listeria monocytogenes str. 4b F2365	RNase HI	NC_002973	1934818-1935219	direct	1-133	A
Geobacillus kaustophilus HTA426	RNase HI	NC_006510	1372704-1373369	direct	71-221	A
Bacillus clausii KSM-K16	RNase HI'	NC_006582	1399495-1400094	direct	74-199	B'
Bacillus halodurans C-125	RNase HI'	NC_002570	933504-934094	direct	69-196	B'
