# Supplementary material for: Evolution of ribonuclease H genes in prokaryotes to avoid inheritance of redundant genes
Source: BMC Evol Biol. 2007 Jul 31;7:128. doi: 10.1186/1471-2148-7-128 (PMC1950709; doi:10.1186/1471-2148-7-128)
Supplement: Additional file 3 — List of RNase H genes from the species whose genomes encode dsRHbd sequences. [file 1471-2148-7-128-S3.pdf]

Additional file 3: List of RNase H genes from the species whose genomes encode dsRHbd sequences. ORF numbers indicate the genomic positions of the genes encoding RNase H. Domain numbers indicate the amino acid positions relative to the start of each protein sequences. The RNase H combination refers to the groups defined in Figure 1. Apostrophes (i.e., B') represent the presence of dsRHbd. Species names followed by an asterisk indicate the presence of multiple RNase HI genes.

Species	Type	Accession No.	ORF	Direction	Domain	Combination
**Euryarchaeota**						
Methanococcus maripaludis	RNase HI'	NC_005791	832383-832988	complement	65-201	B'
	RNase HII	NC_005791	1355645-1356373	direct	24-225	
**Bacteroidetes**						
Bacteroides fragilis NCTC 9343	RNase HI'	NC_003228	207338-207967	complement	79-209	B'
	RNase HII	NC_003228	263354-263959	direct	13-191	
Bacteroides fragilis YCH46	RNase HI'	NC_006347	253987-254616	complement	79-209	B'
	RNase HII	NC_006347	318671-319276	direct	13-191	
Bacteroides thetaiotaomicron	RNase HI'	NC_004663	4371820-4372455	complement	81-211	B'
	RNase HII	NC_004663	4395851-4396453	direct	13-191	
Porphyromonas gingivalis	RNase HI'	NC_002950	1292573-1293223	direct	85-216	B'
	RNase HII	NC_002950	785748-786353	direct	14-192	
**Cyanobacteria**						
Gloeobacter violaceus	RNase HI'	NC_005125	3808044-3808685	complement	1-138	B'
	RNase HII	NC_005125	1622346-1622942	direct	2-178	
**Firmicutes**						
*Bacillus clausii**	RNase HI'	NC_006582	1399495-1400094	direct	74-199	B'
	RNase HI	NC_006582	2129695-2130096	complement	1-133	
	RNase HII	NC_006582	2409239-2410018	complement	72-248	
*Bacillus halodurans**	RNase HI'	NC_002570	933504-934094	direct	69-196	B'
	RNase HI	NC_002570	1842852-1843259	direct	1-135	
	RNase HI	NC_002570	2405735-2406187	direct	1-150	
	RNase HII	NC_002570	2594397-2595188	complement	77-253	
*Clostridium acetobutylicum**	RNase HI	NC_003030	2006983-2007720	complement	4-137	B'
	RNase HI'	NC_003030	2659515-2660237	complement	103-238	
	RNase HII	NC_003030	1907294-1908058	direct	70-252	
Clostridium perfringens	RNase HI'	NC_003366	1707913-1708542	complement	70-207	B'
	RNase HII	NC_003366	1985691-1986509	complement	90-270	
Clostridium tetani	RNase HI'	NC_004557	2281472-2282092	complement	68-203	B'
	RNase HII	NC_004557	1343746-1344561	direct	87-269	
Desulfitobacterium hafniense	RNase HI'	NC_007907	2075150-2075770	complement	71-203	B'
	RNase HII	NC_007907	2957362-2958144	complement	76-253	
Lactobacillus acidophilus	RNase HI'	NC_006814	116459-117205	direct	91-245	B'
	RNase HII	NC_006814	952980-953732	direct	69-245	
Lactobacillus delbrueckii	RNase HI'	NC_008054	146497-147264	direct	93-253	B'
	RNase HII	NC_008054	1080827-1081597	complement	70-246	
Lactobacillus johnsonii	RNase HI'	NC_005362	118550-119281	direct	86-240	B'
	RNase HII	NC_005362	1011011-1011763	direct	69-245	
*Lactobacillus plantarum**	RNase HI'	NC_004567	2310574-2311470	direct	70-225	B'
	RNase HI	NC_004567	1609565-1609951	complement	1-128	
	RNase HII	NC_004567	1674735-1675502	complement	73-249	
*Lactobacillus salivarius**	RNase HI'	NC_007929	459722-460381	direct	59-216	B'
	RNase HI	NC_007929	858179-858565	complement	2-128	
	RNase HII	NC_007929	763125-763892	direct	73-249	
Mesoplasma florum	RNase HI'	NC_006055	559864-560484	complement	63-199	B'
	RNase HII	NC_006055	633730-634356	complement	23-200	
Mycoplasma capricolum	RNase HI'	NC_007633	382602-383222	direct	63-198	B'
	RNase HII	NC_007633	651769-652392	complement	21-199	
Mycoplasma mycoides	RNase HI'	NC_005364	375791-376408	direct	62-198	B'
	RNase HII	NC_005364	483996-484619	direct	21-199	
Mycoplasma penetrans	RNase HI'	NC_004432	1267458-1268120	complement	73-216	B'
	RNase HII	NC_004432	1333337-1333993	complement	31-210	

Additional file 3 (Continued)

Species	Type	Accession No.	ORF	Direction	Domain	Combination
**Fusobacteria**						
Fusobacterium nucleatum	RNase HI'	NC_003454	1651474-1652124	complement	70-216	B'
	RNase HII	NC_003454	2019445-2020092	complement	22-205	
**Deltaproteobacteria**						
*Bdellovibrio bacteriovorus**	RNase HI	NC_005363	2044076-2044537	complement	14-151	B'
	RNase HI'	NC_005363	3036447-3037238	complement	18-164	
	RNase HII	NC_005363	2025670-2026335	complement	39-217	
Desulfotalea psychrophila	RNase HI'	NC_006138	1023218-1024003	direct	105-247	B'
	RNase HII	NC_006138	3163253-3163930	direct	31-211	
*Myxococcus xanthus**	RNase HI	NC_008095	7095320-7095763	direct	14-147	B'
	RNase HI'	NC_008095	2628106-2628873	complement	3-154	
	RNase HII	NC_008095	2994879-2995760	complement	84-262	
**Gammaproteobacteria**						
Colwellia psychrerythraea	RNase HI'	NC_003910	1743847-1744665	complement	98-247	B'
	RNase HII	NC_003910	1612598-1613251	direct	19-208	
*Photobacterium profundum**	RNase HI'	NC_006370	2161121-2161870	complement	77-225	B'
	RNase HI	NC_006370	3350860-3351399	direct	25-166	
	RNase HII	NC_006370	3387343-3387948	complement	17-194	
*Saccharophagus degradans**	RNase HI'	NC_007912	82187-82945	complement	81-228	B'
	RNase HI	NC_007912	4381311-4381808	complement	1-153	
	RNase HII	NC_007912	3277856-3278455	complement	12-189	
*Shewanella denitrificans**	RNase HI'	NC_007954	880428-881219	direct	91-239	B'
	RNase HI	NC_007954	2395224-2395703	direct	5-145	
	RNase HII	NC_007954	1822231-1822875	direct	27-208	
**Spirochaetes**						
Borrelia burgdorferi	RNase HI'	NC_001318	897096-897740	complement	74-212	B'
	RNase HII	NC_001318	45446-45991	direct	3-181	
Borrelia garinii	RNase HI'	NC_006156	899069-899668	complement	58-196	B'
	RNase HII	NC_006156	45306-45851	direct	3-181	
**Thermotogae**						
Thermotoga maritima	RNase HI'	NC_000853	1322788-1323459	complement	63-197	B'
	RNase HII	NC_000853	933029-933745	complement	15-188	
