# Supplementary material for: Evolution of ribonuclease H genes in prokaryotes to avoid inheritance of redundant genes
Source: BMC Evol Biol. 2007 Jul 31;7:128. doi: 10.1186/1471-2148-7-128 (PMC1950709; doi:10.1186/1471-2148-7-128)
Supplement: Additional file 4 — RNase HI sequences from 12 species in the gammaproteobacteria used for the Bayesian phylogenetic analysis. [file 1471-2148-7-128-S4.pdf]

Additional file 4: RNase HI sequences from 12 species in the gammaproteobacteria used for the phylogenetic analysis. ORF numbers indicate the genomic positions of the genes encoding RNase H. Domain numbers indicate the amino acid positions relative to the start of each protein sequences. . The RNase H combination refers to the groups defined in Figure 1. Apostrophes (i.e., B') represent the presence of dsRHbd.

Species	Type	Accession No.	ORF	Direction	Domain	Combination
Colwellia psychrerythraea 34H	RNase HI'	NC 003910	1743847-1744665	complement	98-247	B'
Escherichia coli K12	RNase HI	NC 000913	235535-236002	complement	2-142	B
Idiomarina loihiensis L2TR	RNase HI	NC 006512	1822411-1822884	direct	5-144	B
Photobacterium profundum SS9	RNase HI'	NC 006370	2161121-2161870	complement	77-225	B'
	RNase HI	NC_006370	3350860-3351399	direct	25-166	
Pseudoalteromonas atlantica T6c	RNase HI	NC 008228	2880192-2880653	direct	1-141	B
	RNase HI	NC_008228	4074405-4074896	complement	1-159	
Pseudoalteromonas haloplanktis TAC125	RNase HI	NC 007481	2068513-2068977	direct	2-142	B
Saccharophagus degradans 2-40	RNase HI'	NC 007912	82187-82945	complement	81-228	B'
Shewanella denitrificans OS217	RNase HI'	NC 007954	880428-881219	direct	91-239	B'
	RNase HI	NC_007954	2395224-2395703	direct	5-145	
Vibrio cholerae O1 biovar eltor str. N16961	RNase HI	NC 002505	530684-531124	direct	1-144	B
	RNase HI	NC_002505	2388824-2389294	direct	2-142	
Vibrio fischeri ES114	RNase HI	NC 006840	2171052-2171525	direct	6-146	B
	RNase HI	NC_006841	598303-598749	direct	4-147	
Vibrio parahaemolyticus RIMD 2210633	RNase HI	NC 004603	672008-672505	direct	15-162	B
	RNase HI	NC_004603	2403675-2404139	direct	2-142	
	RNase HI	NC_004605	429180-429689	direct	15-162	
Vibrio vulnificus YJ016	RNase HI	NC 005139	503675-504112	direct	1-144	B
	RNase HI	NC_005139	2560072-2560539	direct	2-142	
